# Supplementary material for: Insights into the molecular basis of tick-borne encephalitis from multiplatform metabolomics
Source: PLoS Negl Trop Dis. 2021 Mar 10;15(3):e0009172. doi: 10.1371/journal.pntd.0009172 (PMC7984639; doi:10.1371/journal.pntd.0009172)
Supplement: S1 Table — (DOCX) [file pntd.0009172.s001.docx]

| Characteristics | Normal range | Forest encephalitis | Healthy individuals |
| --- | --- | --- | --- |
| No. of subjects |  | 50(31AP/19RP) | 39 |
| Gender(M/F) |  | 39/11 | 19/20 |
| Age |  | 25–68 | 22–59 |
| IgG: IgM | Positive/Negative | Positive | Negative |
|  |  |  |  |
