# Supplementary material for: Insights into the molecular basis of tick-borne encephalitis from multiplatform metabolomics
Source: PLoS Negl Trop Dis. 2021 Mar 10;15(3):e0009172. doi: 10.1371/journal.pntd.0009172 (PMC7984639; doi:10.1371/journal.pntd.0009172)
Supplement: S2 Table — (DOCX) [file pntd.0009172.s002.docx]

|  | **Metabolomics** | | | | **Lipidomics** | |
| --- | --- | --- | --- | --- | --- | --- |
|  | Low polarity | | High polarity | |  |  |
| **Column** | HSS T3 column | | BEH Amide column | | Phenomenex Kinetex C18 column | |
| **Flow** | 0.3 mL/min | | | | | |
| **Injection volume** | 5 μL | | | | 1 μL | |
| **Mobile Phase** | H2O (0.1%FA) | Methanol:  acetonitrile  1:1 (V:V) | H2O (25mM CH3COONH4  +25mM NH4OH) | acetonitrile | H2O/MeOH/ACN=1:1:1 (10mM NH4Ac) | IPA |
| **Elution gradient** | 0–2 min, 5%–5%; 2–5 min, 5%–70%; 5–14 min, 70%–90%; 14–16 min, 90%–100%; 16–20 min, 100%–100%; 20–20.1 min, 100%–5%; 20.1–25 min, 5%–5% | | 0–1 min, 95%–95%; 1–14 min, 95%–65%; 14–16 min, 65%–40%; 16–18 min, 40%–40%;  18–18.1 min, 40%–95%; 18.1–23 min, 95%–95% | | 0–3 min, 20%–40%; 3–6 min, 40%–60%; 6–13 min, 60%–80%; 13–17 min, 80%–100%; 17–19 min, 100%–100%; 19–19.1 min, 100%–20%; 19.1–23 min, 20%–20% | |
| **MS parameters** | CUR: 35；GS1: 60；GS2:60; ISVF:5500/–4500; TEM: 550; DP: 80; CE: 35 ±15 | | | | CUR: 40; GS1: 55; GS2: 55; ISVF: 5500/–4500;  TEM: 500; DP: 60; CE:35 ± 15 | |
