# Supplementary material for: Insights into the molecular basis of tick-borne encephalitis from multiplatform metabolomics
Source: PLoS Negl Trop Dis. 2021 Mar 10;15(3):e0009172. doi: 10.1371/journal.pntd.0009172 (PMC7984639; doi:10.1371/journal.pntd.0009172)
Supplement: S3 Table — (DOCX) [file pntd.0009172.s003.docx]

**Data interpretation**

**Amino acid**

**Energy metabolism**

[**phospholipid**](javascript:;)**/ TAG**

**(Metabolomics)144/(Lipidomics)14metabolites with significant differences between TBEV group and control group**

**Model establishment**

**Discriminant model**

**T3 column**

**Hilic column**

**Phenomenex Kinetex C18 column cccolumncolumncolumn**

**Lipidomics**

**Metabolomics**

**RP n=39, AP n=11**

**LC-ESI-MS analysis**

**Control serum samples n=39**

**TBEV patients serum samples n=50**
