# Supplementary material for: Insights into the molecular basis of tick-borne encephalitis from multiplatform metabolomics
Source: PLoS Negl Trop Dis. 2021 Mar 10;15(3):e0009172. doi: 10.1371/journal.pntd.0009172 (PMC7984639; doi:10.1371/journal.pntd.0009172)
Supplement: S4 Table — (DOCX) [file pntd.0009172.s004.docx]

| **Metabolite** | **AUROC** |
| --- | --- |
| 910-EpOME | 1 |
| 9,10-DHOME | 0.99 |
| Alpha-linolenic acid | 0.99 |
| Azelaic acid | 0.99 |
| Glucose 1-phosphate | 0.99 |
| Glucose 6-phosphate | 0.99 |
| Mannose 6-phosphate | 0.99 |
| D-Glutamine | 0.99 |
| L-Glutamine | 0.99 |
| L-Glutamic acid | 0.99 |
| 16-Oxo-palmitate | 0.97 |
| Methionine sulfoxide | 0.97 |
| DL-Methionine sulfoxide | 0.97 |
| Leukotriene A4 | 0.96 |
| 4-Guanidinobutanoic acid | 0.95 |
| Acetaminophen | 0.94 |
| Glycochenodeoxycholate | 0.94 |
| L-Aspartyl-L-phenylalanine | 0.93 |
| Bilirubin | 0.93 |
| Hydrocinnamic acid | 0.92 |
| Alpha-ketoisovaleric acid | 0.91 |
| L-Cystine | 0.90 |
