# Supplementary material for: Insights into the molecular basis of tick-borne encephalitis from multiplatform metabolomics
Source: PLoS Negl Trop Dis. 2021 Mar 10;15(3):e0009172. doi: 10.1371/journal.pntd.0009172 (PMC7984639; doi:10.1371/journal.pntd.0009172)
Supplement: S5 Table — (DOCX) [file pntd.0009172.s005.docx]

| **Acquisition methods:** T3 column_positive mode | | | | | | | |
| --- | --- | --- | --- | --- | --- | --- | --- |
| **NO.** | **Component Name** | **Retention Time** | **Formula** | **Adduct / Charge** | **Precursor Mass** | **Found At Mass** | **Mass Error (ppm)** |
| 1 | D-Alpha-aminobutyric acid | 1.27 | C4H9NO2 | [M+H]+ | 104.071 | 104.0709 | 3.1 |
| 2 | Dimethylglycine | 1.27 | C4H9NO2 | [M+H]+ | 104.071 | 104.0709 | 3.1 |
| 3 | L-Alpha-aminobutyric acid | 1.27 | C4H9NO2 | [M+H]+ | 104.071 | 104.0709 | 3.1 |
| 4 | Creatinine | 1.24 | C4H7N3O | [M+H]+ | 114.066 | 114.0661 | -0.4 |
| 5 | L-Proline | 1.26 | C5H9NO2 | [M+H]+ | 116.071 | 116.0707 | 1.2 |
| 6 | L-Valine | 1.21 | C5H11NO2 | [M+H]+ | 118.086 | 118.0864 | 1.2 |
| 7 | L-Homoserine | 1.2 | C4H9NO3 | [M+H]+ | 120.066 | 120.0652 | -2.5 |
| 8 | L-Threonine | 1.2 | C4H9NO3 | [M+H]+ | 120.066 | 120.0652 | -2.5 |
| 9 | Pyroglutamic acid | 1.39 | C5H7NO3 | [M+H]+ | 130.05 | 130.0497 | -1.6 |
| 10 | Pyrrolidonecarboxylic acid | 1.39 | C5H7NO3 | [M+H]+ | 130.05 | 130.0497 | -1.6 |
| 11 | Pipecolic acid | 1.51 | C6H11NO2 | [M+H]+ | 130.086 | 130.0864 | 0.9 |
| 12 | Creatine | 1.25 | C4H9N3O2 | [M+H]+ | 132.077 | 132.0767 | -0.3 |
| 13 | L-Leucine | 2.59 | C6H13NO2 | [M+H]+ | 132.102 | 132.102 | 0.9 |
| 14 | L-Alloisoleucine | 1.55 | C6H13NO2 | [M+H]+ | 132.102 | 132.1021 | 1.4 |
| 15 | L-Isoleucine | 1.55 | C6H13NO2 | [M+H]+ | 132.102 | 132.1021 | 1.4 |
| 16 | L-Norleucine | 1.55 | C6H13NO2 | [M+H]+ | 132.102 | 132.1021 | 1.4 |
| 17 | Indoxyl | 5.1 | C8H7NO | [M+H]+ | 134.06 | 134.0607 | 4.7 |
| 18 | Urocanic acid | 1.5 | C6H6N2O2 | [M+H]+ | 139.05 | 139.05 | -1.6 |
| 19 | 4-Guanidinobutanoic acid | 1.47 | C5H11N3O2 | [M+H]+ | 146.092 | 146.0923 | -0.9 |
| 20 | D-Glutamine | 1.19 | C5H10N2O3 | [M+H]+ | 147.076 | 147.0761 | -2.3 |
| 21 | L-Glutamine | 1.19 | C5H10N2O3 | [M+H]+ | 147.076 | 147.0761 | -2.3 |
| 22 | D-Lysine | 1.05 | C6H14N2O2 | [M+H]+ | 147.113 | 147.1128 | -0.1 |
| 23 | L-Lysine | 1.05 | C6H14N2O2 | [M+H]+ | 147.113 | 147.1128 | -0.1 |
| 24 | L-Glutamic acid | 1.2 | C5H9NO4 | [M+H]+ | 148.06 | 148.06 | -2.9 |
| 25 | Cinnamic acid | 1.59 | C9H8O2 | [M+H]+ | 149.06 | 149.0596 | -0.9 |
| 26 | trans-Cinnamic acid | 1.59 | C9H8O2 | [M+H]+ | 149.06 | 149.0596 | -0.9 |
| 27 | L-Methionine | 1.54 | C5H11NO2S | [M+H]+ | 150.058 | 150.058 | -2 |
| 28 | 4-Ethylbenzoic acid | 6.27 | C9H10O2 | [M+H]+ | 151.075 | 151.075 | -2.5 |
| 29 | L-Histidine | 1.5 | C6H9N3O2 | [M+H]+ | 156.077 | 156.0766 | -1 |
| 30 | DL-2-Aminooctanoic acid | 5.06 | C8H17NO2 | [M+H]+ | 160.133 | 160.1328 | -2.2 |
| 31 | L-Carnitine | 1.21 | C7H15NO3 | [M+H]+ | 162.112 | 162.1123 | -0.8 |
| 32 | L-Phenylalanine | 1.6 | C9H11NO2 | [M+H]+ | 166.086 | 166.0863 | 0.2 |
| 33 | Perillic acid | 6.83 | C10H14O2 | [M+H]+ | 167.107 | 167.1063 | -2 |
| 34 | L-Arginine | 1.18 | C6H14N4O2 | [M+H]+ | 175.119 | 175.1187 | -1.5 |
| 35 | Indoleacetic acid | 5.8 | C10H9NO2 | [M+H]+ | 176.071 | 176.0707 | 0.6 |
| 36 | Citrulline | 1.2 | C6H13N3O3 | [M+H]+ | 176.103 | 176.1026 | -1.9 |
| 37 | Cotinine | 1.54 | C10H12N2O | [M+H]+ | 177.102 | 177.1019 | -2 |
| 38 | L-Tyrosine | 1.54 | C9H11NO3 | [M+H]+ | 182.081 | 182.0811 | -0.3 |
| 39 | o-Tyrosine | 1.54 | C9H11NO3 | [M+H]+ | 182.081 | 182.0811 | -0.3 |
| 40 | Indoleacrylic acid | 4.76 | C11H9NO2 | [M+H]+ | 188.071 | 188.0706 | -0.2 |
| 41 | 3-Indolepropionic acid | 6.03 | C11H11NO2 | [M+H]+ | 190.086 | 190.0864 | 0.6 |
| 42 | 5-Hydroxyindoleacetic acid | 1.7 | C10H9NO3 | [M+H]+ | 192.066 | 192.0654 | -0.6 |
| 43 | L-Acetylcarnitine | 1.53 | C9H17NO4 | [M+H]+ | 204.123 | 204.1232 | 0.7 |
| 44 | L-Tryptophan | 4.83 | C11H12N2O2 | [M+H]+ | 205.097 | 205.097 | -0.7 |
| 45 | Indolelactic acid | 5.53 | C11H11NO3 | [M+H]+ | 206.081 | 206.0816 | 2.1 |
| 46 | Pantothenic acid | 4.68 | C9H17NO5 | [M+H]+ | 220.118 | 220.1173 | -2.9 |
| 47 | Alpha-Linolenic acid | 8.96 | C18H30O2 | [M+H]+ | 279.232 | 279.2318 | -0.2 |
| 48 | L-Aspartyl-L-phenylalanine | 4.83 | C13H16N2O5 | [M+H]+ | 281.113 | 281.1134 | 0.7 |
| 49 | 1-Methyladenosine | 1.38 | C11H15N5O4 | [M+H]+ | 282.12 | 282.1195 | -0.7 |
| 50 | Cortisol | 5.94 | C21H30O5 | [M+H]+ | 363.217 | 363.2167 | 0.2 |
| 51 | N-Methyl-a-aminoisobutyric acid | 1.21 | C5H11NO2 | [M+H]+ | 118.086 | 118.0864 | 1.2 |
| 52 | Sphinganine | 7.6 | C18H39NO2 | [M+H]+ | 302.305 | 302.3055 | 0.4 |
| 53 | Ribothymidine | 1.31 | C10H14N2O6 | [M+H]+ | 259.092 | 259.0927 | 0.7 |
| 54 | Cortisone | 5.96 | C21H28O5 | [M+H]+ | 361.201 | 361.201 | 0.1 |
| 55 | Hypoxanthine | 1.54 | C5H4N4O | [M+H]+ | 137.046 | 137.0456 | -1.5 |
| 56 | Uric acid | 1.52 | C5H4N4O3 | [M+H]+ | 169.036 | 169.0357 | 0.7 |
| 57 | DL-Citrulline | 1.2 | C6H13N3O3 | [M+H]+ | 176.103 | 176.1026 | -1.9 |
| 58 | L-Kynurenine | 1.71 | C10H12N2O3 | [M+H]+ | 209.092 | 209.0921 | 0.1 |
| 59 | Inosine | 1.54 | C10H12N4O5 | [M+H]+ | 269.088 | 269.088 | -0.1 |
| 60 | Guanosine | 1.54 | C10H13N5O5 | [M+H]+ | 284.099 | 284.0991 | 0.4 |
| 61 | Vitamin A | 14 | C20H30O | [M+H]+ | 287.237 | 287.2371 | 0.7 |
| 62 | Xanthine | 1.54 | C5H4N4O2 | [M+H]+ | 153.041 | 153.0411 | 2.4 |
| 63 | Pantothenate | 4.81 | C9H17NO5CH3CN | [M+H]+ | 261.144 | 261.1445 | 0 |
| 64 | 9(10)-EpOME | 7.16 | C18H32O3 | [2M+K]+ | 631.433 | 631.4328 | -1 |
| 65 | 10,16-Dihydroxyhexadecanoic acid | 8.13 | C16H32O4 | [2M+K]+ | 615.423 | 615.4239 | 1 |
| 66 | 22-Hydroxydocosahexaenoate | 10.03 | C22H32O3 | [M+H]+ | 345.242 | 345.243 | 1.6 |
| 67 | Anthraniloyl-CoA | 5.59 | C28H41N8O17P3S | [M+H]+ | 887.16 | 887.1556 | -4.6 |
| 68 | delta-12-Prostaglandin J2 | 8.57 | C20H30O4 | [M+H]+ | 335.222 | 335.2219 | 0.6 |
| 69 | Latia luciferin | 6.17 | C15H24O2 | [2M+Na]+ | 495.344 | 495.3445 | 0.1 |
| 70 | Leukotriene A4 | 9.58 | C20H30O3 | [2M+H]+ | 637.446 | 637.4488 | 4 |
| 71 | Pyrimidine nucleoside | 1.36 | C9H13N2O4CH3CN | [M+Na]+ | 277.103 | 277.1035 | 0.8 |
| 72 | (4Z,7Z,10Z,13Z,16Z,19Z)-Docosahexaenoic acid | 11.26 | C22H32O2 | [M+H]+ | 329.248 | 329.2479 | 1.1 |
| 73 | (S)-Norcoclaurine | 5.2 | C16H17NO3CH3CN | [M+H]+ | 313.155 | 313.1548 | 0.5 |
| 74 | (5Z,8Z,11Z,14Z,17Z)-Icosapentaenoic acid | 9.79 | C20H30O2 | [M+H]+ | 303.232 | 303.2319 | 0.2 |
| 75 | Linoleate | 9.49 | C18H32O2 | [M+H]+ | 281.248 | 281.247 | -1.9 |
| 76 | Bilirubin | 5.34 | C33H36N4O6 | [M+H]+ | 585.271 | 585.2706 | -0.3 |
| 77 | 8(R)-HPETE | 7.37 | C20H32O4 | [M+Na]+ | 359.219 | 359.2191 | -0.6 |
| 78 | Arachidonate | 11.17 | C20H32O2 | [M+H]+ | 305.248 | 305.2476 | 0.3 |
| 79 | Colneleate | 9.41 | C18H30O3 | [M+Na]+ | 317.209 | 317.209 | 0.8 |
| 80 | Hepoxilin A3 | 7.02 | C20H32O4 | [M+Na]+ | 359.219 | 359.2197 | 1.1 |
| 81 | 12-Keto-leukotriene B4 | 6.86 | C20H30O4 | [M+H]+ | 335.222 | 335.2214 | -0.7 |
| 82 | (5Z,7E,9E,14Z,17Z)-Eicosapentaenoic acid | 13.23 | C20H30O2 | [M+H]+ | 303.232 | 303.2321 | 0.8 |
| 83 | Phytosphingosine | 6.44 | C18H39NO3 | [M+H]+ | 318.3 | 318.3006 | 1 |
| 84 | 3-Ketosphingosine | 8.47 | C18H35NO2 | [M+Na]+ | 320.256 | 320.2562 | 0.6 |
| 85 | Prostaglandin H2 | 7.13 | C20H32O5 | [M+Na]+ | 375.214 | 375.2143 | 0.2 |
| 86 | Sphingosine 1-phosphate | 7.2 | C18H38NO5P | [M+H]+ | 380.256 | 380.2562 | 0.4 |
| 87 | Hexadecanoic acid | 9.92 | C16H32O2CH3CN | [M+Na]+ | 320.256 | 320.2562 | 0.7 |
| 88 | 2-Succinylbenzoyl-CoA | 5.59 | C32H44N7O20P3S | [M+H]+ | 972.165 | 972.1665 | 1.8 |
| 89 | Hexadecenal | 10.1 | C16H30OCH3CN | [M+H]+ | 280.263 | 280.2636 | 0.5 |
| 90 | 5,6-DHET | 9.83 | C20H34O4 | [M+NH4]+ | 356.28 | 356.2787 | -2.4 |
| 91 | (5-L-Glutamyl)-L-amino acid | 1.38 | C8H14N2O5 | [M+H]+ | 219.098 | 219.0977 | 0.6 |
| 92 | Sphinganine 1-phosphate | 7.43 | C18H40NO5P | [M+H]+ | 382.272 | 382.272 | 0.9 |
| 93 | (9Z)-Hexadecenoic acid | 9.27 | C16H30O2CH3CN | [M+H]+ | 296.258 | 296.2582 | -0.7 |
| 94 | Thebaine | 4.79 | C19H21NO3 | [M+Na]+ | 334.141 | 334.142 | 1.9 |
| 95 | Pentadecanal | 12.57 | C15H30OCH3CN | [M+H]+ | 268.263 | 268.2632 | -1.1 |
| 96 | D-Ornithine | 1.1 | C5H12N2O2 | [M+H]+ | 133.097 | 133.0971 | -0.4 |
| 97 | L-Citrulline | 1.19 | C6H13N3O3 | [M+Na]+ | 198.085 | 198.0845 | -1.8 |
| 98 | Choline phosphate | 8.37 | C5H14NO4P | [M+H]+ | 184.073 | 184.0734 | 0.6 |
| 99 | 3-Indoleacrylate | 4.83 | C11H9NO2 | [M+H]+ | 188.071 | 188.0704 | -0.8 |
| 100 | Pidolic acid | 1.92 | C5H7NO3 | [M+H]+ | 130.05 | 130.0499 | 0 |
| 101 | L-Palmitoylcarnitine | 7.17 | C23H45NO4 | [M+K]+ | 438.298 | 438.2977 | -0.8 |
| 102 | Hypoxanthine1 | 1.54 | C5H4N4O | [M+H]+ | 137.046 | 137.0456 | -1.5 |
| 103 | Betaine | 1.2 | C5H11NO2 | [M+Na]+ | 140.068 | 140.0681 | -0.4 |
| 104 | L-Kynurenine1 | 1.71 | C10H12N2O3 | [M+H]+ | 209.092 | 209.0921 | 0.1 |
| 105 | Tauroursodeoxycholic acid | 6.37 | C26H45NO6S | [M+H]+ | 500.304 | 500.3036 | -0.9 |
| 106 | Glycodeoxycholate | 6.78 | C26H43NO5 | [M+H]+ | 450.321 | 450.321 | -0.8 |
| 107 | 20-OH-Leukotriene B4 | 7.45 | C20H32O5HCOOH | [M+K]+ | 437.194 | 437.1932 | -0.9 |
| 108 | Glutaryl-CoA | 5.28 | C26H42N7O19P3SCH3CN | [M+Na]+ | 945.163 | 945.162 | -0.7 |
| 109 | O-Acetylcarnitine | 1.53 | C9H17NO4 | [M+H]+ | 204.123 | 204.1232 | 0.7 |
| 110 | Indole | 4.76 | C8H7N | [M+H]+ | 118.065 | 118.0653 | 1.1 |
| 111 | Prostaglandin G2 | 7.45 | C20H32O6CH3CN | [M+Na]+ | 432.236 | 432.2372 | 3.5 |
| 112 | D-Octopine | 1.38 | C9H18N4O4 | [M+H]+ | 247.14 | 247.1405 | 1.5 |
| 113 | D-Proline | 1.26 | C5H9NO2 | [M+H]+ | 116.071 | 116.0707 | 1.2 |
| 114 | (9Z,12Z,15Z)-Octadecatrienoic acid | 12.68 | C18H30O2 | [M+H]+ | 279.232 | 279.2321 | 0.8 |
| 115 | Biliverdin | 7.11 | C33H34N4O6 | [M+Na]+ | 605.237 | 605.2359 | -1.9 |
| 116 | Resorcinol | 4.82 | C6H6O2CH3CN | [M+H]+ | 152.071 | 152.0706 | -0.2 |
| 117 | Glycochenodeoxycholate 7-sulfate | 6.11 | C26H43NO8S | [M+NH4]+ | 547.305 | 547.3039 | -1.6 |
| 118 | Androstan-3alpha,17beta-diol | 8.2 | C19H32O2 | [M+H]+ | 293.248 | 293.2463 | -4 |
| 119 | 9,10-Dihydroxystearate | 6.66 | C18H36O4CH3CN | [M+H]+ | 358.295 | 358.2952 | 0 |
| 120 | trans-Cinnamoyl beta-D-glucoside | 4.52 | C15H18O7 | [M+NH4]+ | 328.139 | 328.1387 | -1.1 |
| 121 | Thyroxine | 5.95 | C15H11I4NO4 | [M+H]+ | 777.694 | 777.693 | -1.3 |
| 122 | Dehydroepiandrosterone sulfate | 6.25 | C19H28O5S | [M+H]+ | 369.173 | 369.1721 | -2.4 |
| 123 | Amino acid(Arg-) | 1.18 | C6H14N4O2 | [M+H]+ | 175.119 | 175.1187 | -1.5 |
| 124 | L-threo-3-Phenylserine | 2.16 | C9H11NO3 | [M+Na]+ | 204.063 | 204.0629 | -0.9 |
| 125 | Myristoleic acid | 10.99 | C14H26O2 | [M+H]+ | 227.201 | 227.2003 | -1 |
| 126 | 18-Oxooleate | 10.13 | C18H32O3 | [M+H]+ | 297.242 | 297.2426 | 0.7 |
| 127 | 3,4-Dihydroxyphenylacetate | 6.22 | C8H8O4 | [M+H]+ | 169.05 | 169.0489 | -3.5 |
| 128 | Inosine1 | 2.56 | C10H12N4O5 | [M+H]+ | 269.088 | 269.0878 | -0.9 |
| 129 | Skatole | 4.76 | C9H9N | [M+H]+ | 132.081 | 132.0808 | 0.4 |
| 130 | Urate | 1.52 | C5H4N4O3 | [M+H]+ | 169.036 | 169.0357 | 0.7 |
| 131 | N5-Ethyl-L-glutamine | 1.3 | C7H14N2O3 | [M+H]+ | 175.108 | 175.1085 | 4.4 |
| 132 | Sinapine | 5.41 | C16H24NO5HCOOH | [M+K]+ | 395.134 | 395.1342 | 0.2 |
| 133 | CDP-choline | 4.91 | C14H26N4O11P2 | [M+H]+ | 489.115 | 489.1124 | -4.4 |
| 134 | Caffeine | 5.02 | C8H10N4O2 | [M+H]+ | 195.088 | 195.0876 | -0.1 |
| 135 | Indolelactate | 5.53 | C11H11NO3 | [M+H]+ | 206.081 | 206.0816 | 2.1 |
| 136 | trans-Cinnamate | 4.49 | C9H8O2 | [M+H]+ | 149.06 | 149.0596 | -0.5 |
| 137 | Phenylalanine | 4.48 | C9H11NO2 | [M+H]+ | 166.086 | 166.0863 | 0.5 |
| 138 | 17alpha-Hydroxyprogesterone | 7.32 | C21H30O3 | [M+H]+ | 331.227 | 331.2265 | -0.7 |
| 139 | Indole-3-acetate | 5.8 | C10H9NO2 | [M+H]+ | 176.071 | 176.0707 | 0.6 |
| 140 | 3-Oxo-5beta-cholanate | 7.98 | C24H38O3 | [M+H]+ | 375.289 | 375.2886 | -1.9 |
| 141 | Phthalate | 5.2 | C8H6O4 | [M+H]+ | 167.034 | 167.0346 | 4.2 |
| 142 | Hippuric acid | 5.2 | C9H9NO3 | [M+H]+ | 180.066 | 180.0654 | -0.8 |
| 143 | 1,9-Dimethyluric acid | 4.76 | C7H8N4O3 | [M+H]+ | 197.067 | 197.0678 | 4.4 |
| 144 | Riboflavin | 4.96 | C17H20N4O6 | [M+H]+ | 377.146 | 377.1448 | -2.1 |
| 145 | Choline | 1.18 | C5H13NO | [M+H]+ | 104.107 | 104.1075 | 4.7 |
| 146 | Hexadecanal | 9.38 | C16H32OHCOOH | [M+K]+ | 325.214 | 325.2154 | 4.4 |
| 147 | Sinapoyl-CoA | 5.56 | C32H46N7O20P3S | [M+H]+ | 974.18 | 974.1805 | 0.1 |
| 148 | IMP | 4.96 | C10H13N4O8P | [M+H]+ | 349.054 | 349.0549 | 1.4 |
| 149 | cis-9,10-Epoxystearic acid | 8.22 | C18H34O3 | [M+H]+ | 299.258 | 299.2583 | 0.7 |
| 150 | (9Z,11E)-(13S)-13-Hydroperoxyoctadeca-9,11-dienoic acid | 7.08 | C18H32O4 | [M+Na]+ | 335.219 | 335.2189 | -1.2 |
| 151 | Sphingosine | 7.48 | C18H37NO2 | [M+H]+ | 300.29 | 300.2897 | 0 |
| 152 | Phenylacetylglutamine | 5.09 | C13H16N2O4 | [M+Na]+ | 287.1 | 287.0995 | -2.5 |
| 153 | Chenodeoxycholate | 10.9 | C24H40O4 | [M+H]+ | 393.3 | 393.2987 | -3.1 |
| 154 | Pyruvate | 5.1 | C3H4O3CH3CN | [M+H]+ | 130.05 | 130.0502 | 2.4 |
| 155 | Testosterone | 6.05 | C19H28O2 | [M+H]+ | 289.216 | 289.2159 | -1.2 |
| 156 | N5-(L-1-Carboxyethyl)-L-ornithine | 1.59 | C8H16N2O4 | [M+Na]+ | 227.1 | 227.1002 | -0.1 |
| 157 | Leukotriene C4 | 5.2 | C30H47N3O9S | [M+K]+ | 664.266 | 664.2693 | 4.3 |
| 158 | N-Acetyl-L-phenylalanine | 5.95 | C11H13NO3 | [M+H]+ | 208.097 | 208.0961 | -3.5 |
| 159 | Hippurate | 5.2 | C9H9NO3 | [M+H]+ | 180.066 | 180.0654 | -0.8 |
| 160 | (6Z,9Z,12Z)-Octadecatrienoic acid | 8.8 | C18H30O2 | [M+Na]+ | 301.214 | 301.2151 | 4.3 |
| 161 | Apigenin | 6.86 | C15H10O5 | [2M+K]+ | 579.069 | 579.0664 | -4.1 |
| 162 | Prostaglandin F2alpha | 7.4 | C20H34O5CH3CN | [M+H]+ | 396.274 | 396.276 | 3.8 |
| 163 | (Z)-Phenylacetaldehyde oxime | 4.77 | C8H9NO | [M+H]+ | 136.076 | 136.0763 | 4.3 |
| 164 | L-Adrenaline | 4.74 | C9H13NO3 | [M+H]+ | 184.097 | 184.0962 | -3.6 |
| 165 | PC16:016:0 | 15.88 | C40H80NO8P | [M+H]+ | 734.569 | 734.5716 | 2.9 |
| 166 | O-Acetyl-L-serine | 4.57 | C5H9NO4HCOOH | [M+K]+ | 232.022 | 232.022 | 1 |
| **Acquisition methods:** BEH Amide column_positive mode | | | | | | | |
| **NO.** | **Component Name** | **Retention Time** | **Formula** | **Adduct / Charge** | **Precursor Mass** | **Found At Mass** | **Mass Error (ppm)** |
| 1 | D-Alpha-aminobutyric acid | 9.37 | C4H9NO2 | [M+H]+ | 104.071 | 104.0706 | -0.5 |
| 2 | Dimethylglycine | 9.37 | C4H9NO2 | [M+H]+ | 104.071 | 104.0706 | -0.5 |
| 3 | L-Alpha-aminobutyric acid | 9.37 | C4H9NO2 | [M+H]+ | 104.071 | 104.0706 | -0.5 |
| 4 | L-Serine | 11.29 | C3H7NO3 | [M+H]+ | 106.05 | 106.0498 | -0.4 |
| 5 | Uracil | 4.37 | C4H4N2O2 | [M+H]+ | 113.035 | 113.0346 | 0.5 |
| 6 | Creatinine | 5.04 | C4H7N3O | [M+H]+ | 114.066 | 114.0663 | 0.8 |
| 7 | L-Proline | 8.64 | C5H9NO2 | [M+H]+ | 116.071 | 116.0705 | -0.8 |
| 8 | N-Methyl-a-aminoisobutyric acid | 7.69 | C5H11NO2 | [M+H]+ | 118.086 | 118.0863 | 0.2 |
| 9 | L-Homoserine | 10.6 | C4H9NO3 | [M+H]+ | 120.066 | 120.0655 | 0.2 |
| 10 | L-Threonine | 10.6 | C4H9NO3 | [M+H]+ | 120.066 | 120.0655 | 0.2 |
| 11 | Niacinamide | 1.3 | C6H6N2O | [M+H]+ | 123.055 | 123.055 | -2.2 |
| 12 | Pyroglutamic acid | 11.13 | C5H7NO3 | [M+H]+ | 130.05 | 130.05 | 0.8 |
| 13 | Pyrrolidonecarboxylic acid | 11.13 | C5H7NO3 | [M+H]+ | 130.05 | 130.05 | 0.8 |
| 14 | Pipecolic acid | 14.21 | C6H11NO2 | [M+H]+ | 130.086 | 130.0863 | 0.4 |
| 15 | Creatine | 10.06 | C4H9N3O2 | [M+H]+ | 132.077 | 132.0768 | 0.3 |
| 16 | L-Alloisoleucine | 7.8 | C6H13NO2 | [M+H]+ | 132.102 | 132.1018 | -0.5 |
| 17 | L-Isoleucine | 7.8 | C6H13NO2 | [M+H]+ | 132.102 | 132.1018 | -0.5 |
| 18 | L-Norleucine | 7.8 | C6H13NO2 | [M+H]+ | 132.102 | 132.1018 | -0.5 |
| 19 | L-Asparagine | 11.57 | C4H8N2O3 | [M+H]+ | 133.061 | 133.0607 | -0.9 |
| 20 | Indoxyl | 0.79 | C8H7NO | [M+H]+ | 134.06 | 134.0596 | -3.5 |
| 21 | N-Methylnicotinamide | 6.28 | C7H8N2O | [M+H]+ | 137.071 | 137.0708 | -0.8 |
| 22 | Urocanic acid | 3.84 | C6H6N2O2 | [M+H]+ | 139.05 | 139.0501 | -0.9 |
| 23 | D-Glutamine | 11.13 | C5H10N2O3 | [M+H]+ | 147.076 | 147.0764 | -0.1 |
| 24 | L-Glutamine | 11.13 | C5H10N2O3 | [M+H]+ | 147.076 | 147.0764 | -0.1 |
| 25 | D-Lysine | 14.21 | C6H14N2O2 | [M+H]+ | 147.113 | 147.1126 | -1.4 |
| 26 | L-Lysine | 14.21 | C6H14N2O2 | [M+H]+ | 147.113 | 147.1126 | -1.4 |
| 27 | L-Glutamic acid | 11.8 | C5H9NO4 | [M+H]+ | 148.06 | 148.0604 | -0.5 |
| 28 | Hydrocinnamic acid | 1.1 | C9H10O2 | [M+H]+ | 151.075 | 151.0747 | -4.3 |
| 29 | L-Histidine | 14.7 | C6H9N3O2 | [M+H]+ | 156.077 | 156.0768 | 0.2 |
| 30 | Allantoin | 5.26 | C4H6N4O3 | [M+H]+ | 159.051 | 159.0512 | -0.4 |
| 31 | Methionine sulfoxide | 11.33 | C5H11NO3S | [M+H]+ | 166.053 | 166.0533 | 0.1 |
| 32 | L-Phenylalanine | 7.84 | C9H11NO2 | [M+H]+ | 166.086 | 166.0861 | -0.8 |
| 33 | 1-Methylhistidine | 14.46 | C7H11N3O2 | [M+H]+ | 170.092 | 170.0921 | -1.6 |
| 34 | L-Arginine | 13.95 | C6H14N4O2 | [M+H]+ | 175.119 | 175.1189 | -0.3 |
| 35 | Indoleacetic acid | 1.13 | C10H9NO2 | [M+H]+ | 176.071 | 176.0702 | -2.3 |
| 36 | Citrulline | 11.72 | C6H13N3O3 | [M+H]+ | 176.103 | 176.1029 | -0.4 |
| 37 | Hippuric acid | 3.96 | C9H9NO3 | [M+H]+ | 180.066 | 180.0652 | -1.8 |
| 38 | L-Tyrosine | 9.02 | C9H11NO3 | [M+H]+ | 182.081 | 182.0809 | -1.5 |
| 39 | o-Tyrosine | 9.02 | C9H11NO3 | [M+H]+ | 182.081 | 182.0809 | -1.5 |
| 40 | Indoleacrylic acid | 8 | C11H9NO2 | [M+H]+ | 188.071 | 188.0705 | -0.6 |
| 41 | Homo-L-arginine | 13.59 | C7H16N4O2 | [M+H]+ | 189.135 | 189.1344 | -0.8 |
| 42 | 3-Indolepropionic acid | 1.11 | C11H11NO2 | [M+H]+ | 190.086 | 190.0856 | -3.5 |
| 43 | L-Tryptophan | 7.98 | C11H12N2O2 | [M+H]+ | 205.097 | 205.0971 | -0.4 |
| 44 | Pantothenic acid | 4.02 | C9H17NO5 | [M+H]+ | 220.118 | 220.1175 | -2.1 |
| 45 | Glycerophosphocholine | 11.19 | C8H20NO6P | [M+H]+ | 258.11 | 258.1102 | 0.5 |
| 46 | Glucose 1-phosphate | 6.23 | C6H13O9P | [M+H]+ | 261.037 | 261.0372 | 0.7 |
| 47 | Glucose 6-phosphate | 6.23 | C6H13O9P | [M+H]+ | 261.037 | 261.0372 | 0.7 |
| 48 | Mannose 6-phosphate | 6.23 | C6H13O9P | [M+H]+ | 261.037 | 261.0372 | 0.7 |
| 49 | Inosine | 5.97 | C10H12N4O5 | [M+H]+ | 269.088 | 269.0881 | 0.3 |
| 50 | Genistein | 0.69 | C15H10O5 | [M+H]+ | 271.06 | 271.0599 | -0.9 |
| 51 | Alpha-Linolenic acid | 0.97 | C18H30O2 | [M+H]+ | 279.232 | 279.2317 | -0.7 |
| 52 | 1-Methyladenosine | 8.85 | C11H15N5O4 | [M+H]+ | 282.12 | 282.1197 | -0.1 |
| 53 | Cortisol | 1.2 | C21H30O5 | [M+H]+ | 363.217 | 363.2163 | -0.9 |
| 54 | Riboflavin | 6.17 | C17H20N4O6 | [M+H]+ | 377.146 | 377.1454 | -0.3 |
| 55 | 7-Ketocholesterol | 0.93 | C27H44O2 | [M+H]+ | 401.341 | 401.3413 | -0.2 |
| 56 | PC16:016:0 | 4.69 | C40H80NO8P | [M+H]+ | 734.569 | 734.5694 | -0.1 |
| 57 | 2-Piperidinone | 1.24 | C5H9NO | [M+H]+ | 100.076 | 100.0755 | -2.1 |
| 58 | trans-Cinnamic acid | 7.83 | C9H8O2 | [M+H]+ | 149.06 | 149.0596 | -0.9 |
| 59 | 3-Methylhistidine | 14.46 | C7H11N3O2 | [M+H]+ | 170.092 | 170.0921 | -1.6 |
| 60 | 5-Hydroxyindoleacetic acid | 8.04 | C10H9NO3 | [M+H]+ | 192.066 | 192.0653 | -1.2 |
| 61 | L-Acetylcarnitine | 8.53 | C9H17NO4 | [M+H]+ | 204.123 | 204.1225 | -2.4 |
| 62 | Guanosine | 7.54 | C10H13N5O5 | [M+H]+ | 284.099 | 284.0989 | -0.3 |
| 63 | Diethanolamine | 8.05 | C4H11NO2 | [M+H]+ | 106.086 | 106.0862 | -0.4 |
| 64 | Thymine | 3.81 | C5H6N2O2 | [M+H]+ | 127.05 | 127.0501 | -0.8 |
| 65 | 4-Guanidinobutanoic acid | 9.04 | C5H11N3O2 | [M+H]+ | 146.092 | 146.0922 | -1.4 |
| 66 | L-Carnitine | 9.71 | C7H15NO3 | [M+H]+ | 162.112 | 162.1123 | -0.9 |
| 67 | Adenosine | 4.83 | C10H13N5O4 | [M+H]+ | 268.104 | 268.1038 | -0.7 |
| 68 | Sphinganine | 3.52 | C18H39NO2 | [M+H]+ | 302.305 | 302.3051 | -0.7 |
| 69 | Imidazoleacetic acid | 3.81 | C5H6N2O2 | [M+H]+ | 127.05 | 127.0501 | -0.8 |
| 70 | 4-Ethylbenzoic acid | 1.1 | C9H10O2 | [M+H]+ | 151.075 | 151.0747 | -4.3 |
| 71 | Choline [M+H]+ | 5.62 | C5H13NO | [M+H]+ | 104.107 | 104.1069 | -0.4 |
| 72 | L-Leucine | 8.03 | C6H13NO2 | [M+H]+ | 132.102 | 132.1018 | -0.9 |
| 73 | L-Ornithine | 14.39 | C5H12N2O2 | [M+H]+ | 133.097 | 133.0971 | -0.1 |
| 74 | Hypoxanthine | 4.47 | C5H4N4O | [M+H]+ | 137.046 | 137.0457 | -0.5 |
| 75 | L-Methionine | 8.43 | C5H11NO2S | [M+H]+ | 150.058 | 150.0582 | -0.8 |
| 76 | Guanine | 7.55 | C5H5N5O | [M+H]+ | 152.057 | 152.0563 | -2.7 |
| 77 | Xanthine | 5.36 | C5H4N4O2 | [M+H]+ | 153.041 | 153.0405 | -1.2 |
| 78 | Carnitrine | 9.71 | C7H15NO3 | [M+H]+ | 162.112 | 162.1123 | -0.9 |
| 79 | DL-Methionine sulfoxide | 11.33 | C5H11NO3S | [M+H]+ | 166.053 | 166.0533 | 0.1 |
| 80 | Uric acid | 8.91 | C5H4N4O3 | [M+H]+ | 169.036 | 169.0353 | -1.9 |
| 81 | L-Kynurenine | 8.04 | C10H12N2O3 | [M+H]+ | 209.092 | 209.0919 | -1 |
| 82 | Pantothenic Acid1 | 4.02 | C9H17NO5 | [M+H]+ | 220.118 | 220.1175 | -2.1 |
| 83 | L-Cystine; Cys | 15.33 | C6H12N2O4S2 | [M+H]+ | 241.031 | 241.031 | -0.7 |
| 84 | Vitamin B2 | 6.17 | C17H20N4O6 | [M+H]+ | 377.146 | 377.1454 | -0.3 |
| 85 | beta-Alanyl-L-arginine | 9.75 | C9H19N5O3CH3COOH | [M+H]+ | 306.177 | 306.1775 | 1 |
| 86 | 5-L-Glutamyl-taurine | 12.5 | C7H14N2O6S | [M+H]+ | 255.065 | 255.0646 | 0.1 |
| 87 | S-Sulfo-L-cysteine | 9.31 | C3H7NO5S2 | [M+H]+ | 201.984 | 201.9836 | -1.3 |
| 88 | (5-L-Glutamyl)-L-amino acid | 12.42 | C8H14N2O5 | [M+Na]+ | 241.079 | 241.0793 | -0.9 |
| 89 | L-Methionine S-oxide | 11.33 | C5H11NO3S | [M+H]+ | 166.053 | 166.0533 | 0.1 |
| 90 | D-Octopine | 10.12 | C9H18N4O4 | [M+H]+ | 247.14 | 247.1397 | -1.5 |
| 91 | 1-Aminocyclopropane-1-carboxylate | 11.13 | C4H7NO2 | [M+H]+ | 102.055 | 102.055 | 0.8 |
| 92 | L-Methionine1 | 8.43 | C5H11NO2S | [M+H]+ | 150.058 | 150.0582 | -0.8 |
| 93 | Hypoxanthine1 | 4.47 | C5H4N4O | [M+H]+ | 137.046 | 137.0457 | -0.5 |
| 94 | 4-Guanidinobutanoate | 9.04 | C5H11N3O2 | [M+H]+ | 146.092 | 146.0922 | -1.4 |
| 95 | 10-Formyldihydrofolate | 11.11 | C20H21N7O7 | [M+K]+ | 510.113 | 510.114 | 1.2 |
| 96 | Pseudouridine | 6.81 | C9H12N2O6 | [M+H]+ | 245.077 | 245.0767 | -0.6 |
| 97 | (R)-2-Methylmalate | 5.41 | C5H8O5 | [M+NH4]+ | 166.071 | 166.0711 | 0.4 |
| 98 | Urate | 8.91 | C5H4N4O3 | [M+H]+ | 169.036 | 169.0353 | -1.9 |
| 99 | L-Pipecolate | 7.58 | C6H11NO2 | [M+H]+ | 130.086 | 130.0862 | 0 |
| 100 | Indolepyruvate | 8.01 | C11H9NO3 | [M+NH4]+ | 221.092 | 221.0913 | -3.5 |
| 101 | Ergothioneine | 9.43 | C9H15N3O2S | [M+H]+ | 230.096 | 230.0956 | -0.9 |
| 102 | L-Kynurenine1 | 8.1 | C10H12N2O3 | [M+Na]+ | 231.074 | 231.0739 | -0.4 |
| 103 | 5-Hydroxyindoleacetate | 8.04 | C10H9NO3 | [M+H]+ | 192.066 | 192.0653 | -1.2 |
| 104 | Bilirubin | 1.8 | C33H36N4O6 | [M+H]+ | 585.271 | 585.2706 | -0.3 |
| 105 | Sarcosine | 9.99 | C3H7NO2 | [M+H]+ | 90.055 | 90.0551 | 2.1 |
| 106 | D-Ornithine | 14.39 | C5H12N2O2 | [M+H]+ | 133.097 | 133.0971 | -0.1 |
| 107 | N6-Acetyl-L-lysine | 10.27 | C8H16N2O3 | [M+H]+ | 189.123 | 189.1234 | -0.1 |
| 108 | (2R,3S)-2,3-Dimethylmalate | 5.43 | C6H10O5 | [M+NH4]+ | 180.087 | 180.0869 | 1.2 |
| 109 | 4-Coumarate | 9.02 | C9H8O3 | [M+H]+ | 165.055 | 165.0544 | -1.5 |
| 110 | Biliverdin | 1.19 | C33H34N4O6 | [M+H]+ | 583.255 | 583.2546 | -0.9 |
| 111 | O-Acetylcarnitine | 8.52 | C9H17NO4 | [M+Na]+ | 226.105 | 226.1041 | -3.7 |
| 112 | Kyotorphin | 11.88 | C15H23N5O4 | [M+H]+ | 338.182 | 338.1808 | -4.4 |
| 113 | L-threo-3-Phenylserine | 9.02 | C9H11NO3 | [M+H]+ | 182.081 | 182.0809 | -1.5 |
| 114 | Xanthine1 | 5.36 | C5H4N4O2 | [M+H]+ | 153.041 | 153.0405 | -1.2 |
| 115 | Guanidinoacetate | 10.27 | C3H7N3O2 | [M+H]+ | 118.061 | 118.061 | -0.5 |
| 116 | L-Palmitoylcarnitine | 5.17 | C23H45NO4 | [M+Na]+ | 422.324 | 422.3259 | 4.4 |
| 117 | alpha,beta-Didehydrotryptophan | 8.05 | C11H10N2O2CH3CN | [M+H]+ | 244.108 | 244.1075 | -2.2 |
| 118 | 2-Hydroxypyridine | 6.28 | C5H5NOCH3CN | [M+H]+ | 137.071 | 137.0708 | -0.8 |
| 119 | N-Acetylornithine | 10.59 | C7H14N2O3 | [M+H]+ | 175.108 | 175.1076 | -0.5 |
| 120 | D-erythro-3-Methylmalate | 6.55 | C5H8O5 | [M+NH4]+ | 166.071 | 166.0712 | 1.1 |
| 121 | trans-Cinnamate | 7.83 | C9H8O2 | [M+H]+ | 149.06 | 149.0596 | -0.9 |
| 122 | 5-Aminopentanoate | 8.75 | C5H11NO2 | [M+H]+ | 118.086 | 118.0862 | -0.6 |
| 123 | 4-Aminobutanoate | 9.37 | C4H9NO2 | [M+H]+ | 104.071 | 104.0706 | -0.5 |
| 124 | 6-Acetamido-2-oxohexanoate | 6.78 | C8H13NO4CH3CN | [M+H]+ | 229.118 | 229.1184 | 0.4 |
| 125 | 4-Hydroxy-2-oxoglutarate | 6.55 | C5H6O6 | [M+NH4]+ | 180.05 | 180.0506 | 2.1 |
| 126 | D-Proline | 8.64 | C5H9NO2 | [M+H]+ | 116.071 | 116.0705 | -0.8 |
| 127 | Indole-3-acetaldehyde oxime | 5.73 | C10H10N2O | [M+H]+ | 175.087 | 175.0859 | -4.2 |
| 128 | D-Alanyl-D-serine | 11.96 | C6H12N2O4 | [M+Na]+ | 199.069 | 199.0691 | 0.7 |
| 129 | D-allo-Isoleucine | 10 | C6H13NO2 | [M+H]+ | 132.102 | 132.1017 | -1.9 |
| 130 | L-2-Amino-3-oxobutanoic acid | 11.72 | C4H7NO3CH3CN | [M+H]+ | 159.076 | 159.0765 | 0.3 |
| 131 | L-Formylkynurenine | 8.02 | C11H12N2O4 | [M+H]+ | 237.087 | 237.0876 | 2.8 |
| 132 | 2,3-Dimethylmaleate | 6.62 | C6H8O4CH3CN | [M+H]+ | 186.076 | 186.076 | -0.3 |
| 133 | Aminoacetone | 10.61 | C3H7NOCH3COOH | [M+H]+ | 134.081 | 134.0807 | -3.2 |
| 134 | Ornithine | 14.39 | C5H12N2O2 | [M+H]+ | 133.097 | 133.0971 | -0.1 |
| 135 | D-Aspartate | 8.08 | C4H7NO4CH3CN | [M+H]+ | 175.071 | 175.0712 | -0.8 |
| 136 | N6,N6,N6-Trimethyl-L-lysine | 13.38 | C9H20N2O2 | [M+H]+ | 189.16 | 189.1594 | -1.8 |
| 137 | D-Xylose | 3.93 | C5H10O5 | [M+H]+ | 151.06 | 151.0604 | 1.9 |
| 138 | N2-Succinyl-L-arginine | 13.08 | C10H18N4O5 | [M+H]+ | 275.135 | 275.1352 | 0.7 |
| 139 | N-Acetylputrescine | 7.93 | C6H14N2O | [M+H]+ | 131.118 | 131.1185 | 5 |
| 140 | Uridine | 4.35 | C9H12N2O6 | [M+H]+ | 245.077 | 245.0767 | -0.3 |
| 141 | gamma-Amino-gamma-cyanobutanoate | 10.45 | C5H8N2O2 | [M+H]+ | 129.066 | 129.0658 | -0.7 |
| 142 | D-Lysopine | 10.47 | C9H18N2O4 | [M+H]+ | 219.134 | 219.1337 | -1.1 |
| 143 | N(pi)-Methyl-L-histidine | 14.46 | C7H11N3O2 | [M+H]+ | 170.092 | 170.0921 | -1.6 |
| 144 | Betaine aldehyde | 9.71 | C5H11NOCH3COOH | [M+H]+ | 162.112 | 162.1123 | -0.9 |
| 145 | Betaine | 7.69 | C5H11NO2 | [M+K]+ | 156.042 | 156.042 | -0.9 |
| 146 | Phenylalanine | 8.03 | C9H11NO2 | [M+H]+ | 166.086 | 166.086 | -1.6 |
| 147 | N-Hydroxy-L-tryptophan | 6.65 | C11H12N2O3 | [M+K]+ | 259.048 | 259.0467 | -4.7 |
| 148 | Hippurate | 3.96 | C9H9NO3 | [M+H]+ | 180.066 | 180.0652 | -1.8 |
| 149 | Anthranilate | 8.12 | C7H7NO2 | [M+K]+ | 176.011 | 176.0104 | -2.4 |
| 150 | 4-Hydroxy-2-quinolinecarboxylic acid | 6.5 | C10H7NO3 | [M+H]+ | 190.05 | 190.0495 | -2 |
| 151 | N-Amidino-L-aspartate | 11.82 | C5H9N3O4 | [M+H]+ | 176.067 | 176.0664 | -1.1 |
| 152 | 4-Aminobutyraldehyde | 8.06 | C4H9NOCH3COOH | [M+H]+ | 148.097 | 148.0966 | -1.2 |
| 153 | L-Homocitrulline | 11.32 | C7H15N3O3 | [M+Na]+ | 212.101 | 212.1005 | -0.5 |
| 154 | N6-Methyl-L-lysine | 13.52 | C7H16N2O2 | [M+H]+ | 161.128 | 161.1283 | -1 |
| 155 | Nicotine | 4.94 | C10H14N2 | [M+H]+ | 163.123 | 163.1231 | 0.7 |
| 156 | p-Benzoquinone | 8.36 | C6H4O2 | [M+H]+ | 109.028 | 109.0284 | -0.4 |
| 157 | Pidolic acid | 6.28 | C5H7NO3 | [M+NH4]+ | 147.076 | 147.0761 | -2.2 |
| 158 | (E)-4-(Trimethylammonio)but-2-enoate | 9.98 | C7H13NO2 | [M+H]+ | 144.102 | 144.1018 | -0.8 |
| 159 | Phenylacetylglutamine | 6.27 | C13H16N2O4 | [M+H]+ | 265.118 | 265.1183 | 0 |
| 160 | 2-Hydroxy-4-(2-oxo-1,3-dihydro-2H-inden-1-ylidene)but-2-enoic acid | 6.27 | C13H10O4 | [M+NH4]+ | 248.092 | 248.092 | 1 |
| 161 | (S)-Carnitine | 9.33 | C7H15NO3CH3CN | [M+H]+ | 203.139 | 203.1388 | -1.2 |
| 162 | 2-Hydroxy-4-(1-oxo-1,3-dihydro-2H-inden-2-ylidene)-but-2-enoic acid | 6.27 | C13H10O4 | [M+NH4]+ | 248.092 | 248.092 | 1 |
| 163 | 2-Amino-4-oxopentanoic acid | 10.81 | C5H9NO3CH3CN | [M+H]+ | 173.092 | 173.0914 | -3.7 |
| 164 | Calligonine | 5.96 | C12H14N2CH3COOH | [M+H]+ | 247.144 | 247.1441 | -0.1 |
| 165 | Daidzein | 0.71 | C15H10O4 | [M+H]+ | 255.065 | 255.0651 | -0.3 |
| 166 | L-Aspartyl-L-phenylalanine | 10.78 | C13H16N2O5 | [M+H]+ | 281.113 | 281.1127 | -1.9 |
| 167 | Acetaminophen | 1.2 | C8H9NO2 | [M+H]+ | 152.071 | 152.0699 | -4.5 |
| 168 | Cotinine | 1.24 | C10H12N2O | [M+H]+ | 177.102 | 177.102 | -1.2 |
| 169 | 5-Hydroxy-L-tryptophan | 10.78 | C11H12N2O3CH3COOH | [M+H]+ | 281.113 | 281.1127 | -1.9 |
| 170 | 4-O-Dimethylallyl-L-tyrosine | 6.45 | C14H19NO3 | [M+H]+ | 250.144 | 250.1443 | 2.2 |
| 171 | (S)-Lactate | 8.38 | C3H6O3CH3COOH | [M+H]+ | 151.06 | 151.0607 | 4 |
| 172 | L-Citrulline | 10.46 | C6H13N3O3CH3CN | [M+Na]+ | 239.111 | 239.1106 | -3.7 |
| 173 | N-Acetyl-D-glucosaminate | 6.7 | C8H15NO7 | [M+H]+ | 238.092 | 238.0931 | 3.9 |
| 174 | (R)-Malate | 6.57 | C4H6O5 | [M+NH4]+ | 152.055 | 152.0561 | 4.6 |
| 175 | Sphingomyelin | 4.98 | C41H83N2O6P | [M+H]+ | 731.606 | 731.6026 | -4.9 |
| 176 | 2-(Hydroxymethyl)-3-(acetamidomethylene)succinate | 6.36 | C8H11NO6 | [M+H]+ | 218.066 | 218.065 | -4.2 |
| 177 | alpha-Tocopherol | 0.87 | C29H50O2 | [M+H]+ | 431.388 | 431.3862 | -5 |
| 178 | CDP-abequose | 8.59 | C15H25N3O14P2CH3CN | [M+H]+ | 575.115 | 575.1155 | 0.8 |
| 179 | Verapamil | 4.31 | C27H38N2O4 | [M+H]+ | 455.29 | 455.2906 | 0.3 |
| 180 | beta-Alanopine | 8.59 | C6H11NO4 | [M+H]+ | 162.076 | 162.0754 | -4 |
| 181 | Methylguanidine | 9.02 | C2H7N3CH3CN | [M+Na]+ | 137.08 | 137.0792 | -4.5 |
| **Acquisition methods:** BEH Amide column_negative mode | | | | | | | |
| NO. | Component Name | Retention Time | Formula | Adduct / Charge | Precursor Mass | Found At Mass | Mass Error (ppm) |
| 1 | Elaidic acid | 0.96 | C18H34O2 | [M-H]- | 281.249 | 281.2486 | 0 |
| 2 | Oleic acid | 0.96 | C18H34O2 | [M-H]- | 281.249 | 281.2486 | 0 |
| 3 | Vaccenic acid | 0.96 | C18H34O2 | [M-H]- | 281.249 | 281.2486 | 0 |
| 4 | Linoleic acid | 0.97 | C18H32O2 | [M-H]- | 279.233 | 279.2328 | -0.7 |
| 5 | L-Phenylalanine | 7.99 | C9H11NO2 | [M-H]- | 164.072 | 164.0718 | 0.7 |
| 6 | L-Glutamic acid | 12.47 | C5H9NO4 | [M-H]- | 146.046 | 146.0464 | 3.4 |
| 7 | D-Fructose | 8.14 | C6H12O6 | [M-H]- | 179.056 | 179.0563 | 0.9 |
| 8 | Allose | 8.14 | C6H12O6 | [M-H]- | 179.056 | 179.0563 | 0.9 |
| 9 | Alpha-D-Glucose | 8.14 | C6H12O6 | [M-H]- | 179.056 | 179.0563 | 0.9 |
| 10 | D-Fructose | 8.14 | C6H12O6 | [M-H]- | 179.056 | 179.0563 | 0.9 |
| 11 | D-Galactose | 8.14 | C6H12O6 | [M-H]- | 179.056 | 179.0563 | 0.9 |
| 12 | D-Glucose | 8.14 | C6H12O6 | [M-H]- | 179.056 | 179.0563 | 0.9 |
| 13 | D-Tagatose | 8.14 | C6H12O6 | [M-H]- | 179.056 | 179.0563 | 0.9 |
| 14 | L-Sorbose | 8.14 | C6H12O6 | [M-H]- | 179.056 | 179.0563 | 0.9 |
| 15 | Aminocaproic acid | 7.92 | C6H13NO2 | [M-H]- | 130.087 | 130.0878 | 3.2 |
| 16 | Beta-Leucine | 7.92 | C6H13NO2 | [M-H]- | 130.087 | 130.0878 | 3.2 |
| 17 | L-Alloisoleucine | 7.92 | C6H13NO2 | [M-H]- | 130.087 | 130.0878 | 3.2 |
| 18 | L-Leucine | 7.92 | C6H13NO2 | [M-H]- | 130.087 | 130.0878 | 3.2 |
| 19 | D-Glutamine | 11.37 | C5H10N2O3 | [M-H]- | 145.062 | 145.0625 | 4.5 |
| 20 | L-Glutamine | 11.37 | C5H10N2O3 | [M-H]- | 145.062 | 145.0625 | 4.5 |
| 21 | L-Tryptophan | 8.06 | C11H12N2O2 | [M-H]- | 203.083 | 203.0825 | -0.4 |
| 22 | Allantoin | 5.31 | C4H6N4O3 | [M-H]- | 157.037 | 157.0367 | 0 |
| 23 | Cholesterol sulfate | 0.73 | C27H46O4S | [M-H]- | 465.304 | 465.3035 | -2 |
| 24 | 5-Aminopentanoic acid | 8.87 | C5H11NO2 | [M-H]- | 116.072 | 116.0722 | 4 |
| 25 | L-Valine | 8.87 | C5H11NO2 | [M-H]- | 116.072 | 116.0722 | 4 |
| 26 | Uridine | 4.38 | C9H12N2O6 | [M-H]- | 243.062 | 243.0622 | -0.4 |
| 27 | Inosine | 6.01 | C10H12N4O5 | [M-H]- | 267.073 | 267.0734 | -0.2 |
| 28 | Creatinine | 4.74 | C4H7N3O | [M-H]- | 112.052 | 112.052 | 3.6 |
| 29 | Cinnamic acid | 7.97 | C9H8O2 | [M-H]- | 147.045 | 147.0451 | -0.6 |
| 30 | trans-Cinnamic acid | 7.97 | C9H8O2 | [M-H]- | 147.045 | 147.0451 | -0.6 |
| 31 | Azelaic acid | 4.14 | C9H16O4 | [M-H]- | 187.098 | 187.0974 | -0.8 |
| 32 | Pregnenolone sulfate | 0.79 | C21H32O5S | [M-H]- | 395.19 | 395.1892 | -1.4 |
| 33 | Indolelactic acid | 5.48 | C11H11NO3 | [M-H]- | 204.067 | 204.0665 | -0.8 |
| 34 | Pyroglutamic acid | 12.47 | C5H7NO3 | [M-H]- | 128.035 | 128.0358 | 4.1 |
| 35 | Pyrrolidonecarboxylic acid | 12.47 | C5H7NO3 | [M-H]- | 128.035 | 128.0358 | 4.1 |
| 36 | Nervonic acid | 0.93 | C24H46O2 | [M-H]- | 365.343 | 365.3414 | -3.1 |
| 37 | L-3-Phenyllactic acid | 5.03 | C9H10O3 | [M-H]- | 165.056 | 165.0556 | -0.5 |
| 38 | Hydroxyphenyllactic acid | 6.26 | C9H10O4 | [M-H]- | 181.051 | 181.0506 | -0.3 |
| 39 | Citrulline | 11.99 | C6H13N3O3 | [M-H]- | 174.088 | 174.0885 | 0.5 |
| 40 | Acetylglycine | 8.26 | C4H7NO3 | [M-H]- | 116.035 | 116.0358 | 4.3 |
| 41 | Erucic acid | 0.94 | C22H42O2 | [M-H]- | 337.311 | 337.3098 | -4.3 |
| 42 | Sebacic acid | 3.45 | C10H18O4 | [M-H]- | 201.113 | 201.1131 | -0.7 |
| 43 | Dimethylmalonic acid | 8.15 | C5H8O4 | [M-H]- | 131.035 | 131.035 | -0.2 |
| 44 | Glutaric acid | 8.15 | C5H8O4 | [M-H]- | 131.035 | 131.035 | -0.2 |
| 45 | Methylsuccinic acid | 8.15 | C5H8O4 | [M-H]- | 131.035 | 131.035 | -0.2 |
| 46 | Caprylic acid | 1.08 | C8H16O2 | [M-H]- | 143.108 | 143.1078 | 0.6 |
| 47 | Valproic acid | 1.08 | C8H16O2 | [M-H]- | 143.108 | 143.1078 | 0.6 |
| 48 | Chenodeoxycholic acid | 1.21 | C24H40O4 | [M-H]- | 391.285 | 391.2845 | -2.3 |
| 49 | Deoxycholic acid | 1.21 | C24H40O4 | [M-H]- | 391.285 | 391.2845 | -2.3 |
| 50 | Hyodeoxycholic acid | 1.21 | C24H40O4 | [M-H]- | 391.285 | 391.2845 | -2.3 |
| 51 | Ursodeoxycholic acid | 1.21 | C24H40O4 | [M-H]- | 391.285 | 391.2845 | -2.3 |
| 52 | Dodecanedioic acid | 2.55 | C12H22O4 | [M-H]- | 229.145 | 229.1446 | 0.4 |
| 53 | D-Mannose | 8.14 | C6H12O6 | [M-H]- | 179.056 | 179.0563 | 0.9 |
| 54 | 3-Indolepropionic acid | 1.21 | C11H11NO2 | [M-H]- | 188.072 | 188.0716 | -0.8 |
| 55 | Bilirubin | 1.03 | C33H36N4O6 | [M-H]- | 583.256 | 583.2555 | -1.2 |
| 56 | Myristic acid | 1 | C14H28O2 | [M-H]- | 227.202 | 227.2017 | 0.1 |
| 57 | Alpha-ketoisovaleric acid | 1.28 | C5H8O3 | [M-H]- | 115.04 | 115.0406 | 4.7 |
| 58 | Dodecanoic acid | 1.02 | C12H24O2 | [M-H]- | 199.17 | 199.1702 | -0.6 |
| 59 | 2,4-Dihydroxybenzoic acid | 0.72 | C7H6O4 | [M-H]- | 153.019 | 153.0198 | 3.3 |
| 60 | 2,6-Dihydroxybenzoic acid | 0.72 | C7H6O4 | [M-H]- | 153.019 | 153.0198 | 3.3 |
| 61 | Pipecolic acid | 7.82 | C6H11NO2 | [M-H]- | 128.072 | 128.0719 | 1.3 |
| 62 | Pentadecanoic acid | 0.99 | C15H30O2 | [M-H]- | 241.217 | 241.2171 | -0.9 |
| 63 | Capric acid | 1.05 | C10H20O2 | [M-H]- | 171.139 | 171.1393 | 1.5 |
| 64 | Tetradecanedioic acid | 1.21 | C14H26O4 | [M-H]- | 257.176 | 257.1747 | -4.4 |
| 65 | Phthalic acid | 1.28 | C8H6O4 | [M-H]- | 165.019 | 165.0195 | 0.8 |
| 66 | Sucrose | 10.24 | C12H22O11 | [M-H]- | 341.109 | 341.1076 | -3.8 |
| 67 | Trehalose | 10.24 | C12H22O11 | [M-H]- | 341.109 | 341.1076 | -3.8 |
| 68 | Stearic acid | 0.96 | C18H36O2 | [M-H]- | 283.264 | 283.2635 | -2.8 |
| 69 | Mannitol | 7.74 | C6H14O6 | [M-H]- | 181.072 | 181.0719 | 0.6 |
| 70 | Sorbitol | 7.74 | C6H14O6 | [M-H]- | 181.072 | 181.0719 | 0.6 |
| 71 | Taurodeoxycholic acid | 1.27 | C26H45NO6S | [M-H]- | 498.289 | 498.2891 | -0.7 |
| 72 | Methylmalonic acid | 7.86 | C4H6O4 | [M-H]- | 117.019 | 117.0196 | 2.2 |
| 73 | Succinic acid | 7.86 | C4H6O4 | [M-H]- | 117.019 | 117.0196 | 2.2 |
| 74 | 3-Hydroxybenzoic acid | 1.14 | C7H6O3 | [M-H]- | 137.024 | 137.0246 | 1.6 |
| 75 | 4-Hydroxybenzoic acid | 1.14 | C7H6O3 | [M-H]- | 137.024 | 137.0246 | 1.6 |
| 76 | Salicylic acid | 1.14 | C7H6O3 | [M-H]- | 137.024 | 137.0246 | 1.6 |
| 77 | Alpha-Linolenic acid | 0.98 | C18H30O2 | [M-H]- | 277.217 | 277.2172 | -0.3 |
| 78 | Heptadecanoic acid | 0.97 | C17H34O2 | [M-H]- | 269.249 | 269.2487 | 0.2 |
| 79 | Citric acid | 14.06 | C6H8O7 | [M-H]- | 191.02 | 191.0196 | -0.5 |
| 80 | Xanthine | 5.51 | C5H4N4O2 | [M-H]- | 151.026 | 151.0264 | 2 |
| 81 | 3-(4-Hydroxyphenyl)lactate | 6.26 | C9H10O4 | [M-H]- | 181.051 | 181.0506 | -0.3 |
| 82 | DL-Citrulline | 11.99 | C6H13N3O3 | [M-H]- | 174.088 | 174.0885 | 0.5 |
| 83 | DL-Arginine | 15.67 | C6H14N4O2 | [M-H]- | 173.104 | 173.1044 | 0 |
| 84 | D-Pipecolic acid | 7.82 | C6H11NO2 | [M-H]- | 128.072 | 128.0719 | 1.3 |
| 85 | D-Glucuronic acid | 12.05 | C6H10O7 | [M-H]- | 193.035 | 193.0354 | 0.1 |
| 86 | L-Methionine; Met | 8.8 | C5H11NO2S | [M-H]- | 148.044 | 148.0439 | 0.9 |
| 87 | L-Kynurenine | 8.25 | C10H12N2O3 | [M-H]- | 207.078 | 207.0779 | 1.9 |
| 88 | L-Isoleucine | 7.92 | C6H13NO2 | [M-H]- | 130.087 | 130.0878 | 3.2 |
| 89 | α-Linolenic acid | 0.98 | C18H30O2 | [M-H]- | 277.217 | 277.2172 | -0.3 |
| 90 | γ-linolenic acid | 0.98 | C18H30O2 | [M-H]- | 277.217 | 277.2172 | -0.3 |
| 91 | Hypoxanthine | 4.48 | C5H4N4O | [M-H]- | 135.031 | 135.0314 | 1 |
| 92 | Cholic acid | 4.2 | C24H40O5 | [M-H]- | 407.28 | 407.2794 | -2.2 |
| 93 | Galactitol /Mannitol | 7.74 | C6H14O6 | [M-H]- | 181.072 | 181.0719 | 0.6 |
| 94 | Arachidonic acid | 0.96 | C20H32O2 | [M-H]- | 303.233 | 303.2328 | -0.4 |
| 95 | phthalic acid1 | 1.28 | C8H6O4 | [M-H]- | 165.019 | 165.0195 | 0.8 |
| 96 | Uridine1 | 4.38 | C9H12N2O6 | [M-H]- | 243.062 | 243.0622 | -0.4 |
| 97 | Uric acid | 9.96 | C5H4N4O3 | [M-H]- | 167.021 | 167.0214 | 1.8 |
| 98 | D-Glucose1 | 8.14 | C6H12O6 | [M-H]- | 179.056 | 179.0563 | 0.9 |
| 99 | Glycoursodeoxycholic acid | 6.37 | C26H43NO5 | [M-H]- | 448.307 | 448.3064 | -0.9 |
| 100 | Methylmalonic Acid1 | 7.86 | C4H6O4 | [M-H]- | 117.019 | 117.0196 | 2.2 |
| 101 | Guanosine1 | 7.57 | C10H13N5O5 | [M-H]- | 282.084 | 282.0839 | -1.7 |
| 102 | Salicylic Acid1 | 1.14 | C7H6O3 | [M-H]- | 137.024 | 137.0246 | 1.6 |
| 103 | L-Asparagine | 11.92 | C4H8N2O3 | [M-H]- | 131.046 | 131.0466 | 2.9 |
| 104 | N-Acetyllactosamine | 6.37 | C14H25NO11 | [M]- | 383.143 | 383.1419 | -3.6 |
| 105 | Glycocholate | 7.74 | C26H43NO6 | [M-H]- | 464.302 | 464.3012 | -1.2 |
| 106 | N-Acetylneuraminate | 5.97 | C11H19NO9 | [M+AcO-H]- | 368.12 | 368.1214 | 4.2 |
| 107 | 3-Hexenal | 2.51 | C6H10O | [M+AcO-H]- | 157.087 | 157.0874 | 2.2 |
| 108 | (R)-4'-Phosphopantothenoyl-L-cysteine | 6.73 | C12H23N2O9PS | [M-H]- | 401.079 | 401.0772 | -4.2 |
| 109 | (9Z)-Hexadecenoic acid | 4.42 | C16H30O2 | [M-H]- | 253.217 | 253.218 | 2.9 |
| 110 | D-Allose | 3.94 | C6H12O6NH3 | [M+Cl]- | 232.059 | 232.0602 | 3.8 |
| 111 | Pseudouridine | 4.38 | C9H12N2O6 | [M-H]- | 243.062 | 243.0622 | -0.4 |
| 112 | S-(L-Histidin-5-yl)-L-cysteine S-oxide | 5.35 | C9H14N4O5S | [M+K-2H]- | 327.017 | 327.017 | -0.2 |
| 113 | Sinapine | 5.8 | C16H24NO5 | [M+K-2H]- | 347.114 | 347.1149 | 2.5 |
| 114 | Pimelate | 3.12 | C7H12O4 | [M-H]- | 159.066 | 159.0662 | -0.3 |
| 115 | (11E)-Octadecenoic acid | 4.7 | C18H34O2 | [M-H]- | 281.249 | 281.248 | -2.1 |
| 116 | Urate | 2.64 | C5H4N4O3 | [M-H]- | 167.021 | 167.0213 | 1.2 |
| 117 | 3-(4-Hydroxyphenyl)lactate1 | 2.96 | C9H10O4 | [M-H]- | 181.051 | 181.0502 | -2.2 |
| 118 | 1-O-[2-(L-Cysteinamido)-2-deoxy-alpha-D-glucopyranosyl]-1D-myo-inositol | 8.03 | C15H28N2O11SNH3 | [M-H]- | 460.161 | 460.1605 | -0.4 |
| 119 | 1,7-Dimethyluric acid | 3.64 | C7H8N4O3 | [M-H]- | 195.052 | 195.0526 | 1.4 |
| 120 | 6-Tuliposide B | 5.4 | C11H18O9NH3 | [M-H]- | 310.114 | 310.1146 | 0.7 |
| 121 | N-(Carboxymethyl)-D-alanine | 2.2 | C5H9NO4 | [M-H2O-H]- | 128.035 | 128.0359 | 4.9 |
| 122 | D-Glutamate | 2.2 | C5H9NO4 | [M-H2O-H]- | 128.035 | 128.0359 | 4.9 |
| 123 | (S)-Allantoin | 2.45 | C4H6N4O3 | [M-H2O-H]- | 139.026 | 139.0263 | 0.9 |
| 124 | 2-Acetolactate | 1.99 | C5H8O4 | [M-H2O-H]- | 113.024 | 113.0239 | -4.8 |
| 125 | L-Threonine | 10.79 | C4H9NO3 | [M-H]- | 118.051 | 118.0515 | 4.4 |
| 126 | Undecanoic acid | 1.03 | C11H22O2 | [M-H]- | 185.155 | 185.1553 | 3.5 |
| 127 | L-Histidine | 13.9 | C6H9N3O2 | [M-H]- | 154.062 | 154.0624 | 1 |
| 128 | Gluconic acid | 3.58 | C6H12O7 | [M-H]- | 195.051 | 195.0511 | 0.1 |
| 129 | 5-Hydroxyferulic acid methyl ester | 3.73 | C10H10O5 | [M-H]- | 209.046 | 209.0459 | 1.7 |
| 130 | gamma-Amino-gamma-cyanobutanoate | 2.2 | C5H8N2O2 | [M-H]- | 127.051 | 127.0518 | 3.9 |
| 131 | N-Acetylornithine | 2.9 | C7H14N2O3 | [M-H]- | 173.093 | 173.0931 | -0.4 |
| 132 | Phosphatidylethanolamine | 13.77 | C41H82NO8P | [M+AcO-H]- | 806.592 | 806.5897 | -2.4 |
| 133 | Methylmalonate | 2.15 | C4H6O4 | [M-H]- | 117.019 | 117.0191 | -1.8 |
| 134 | 2,5-Dioxopentanoate | 2.16 | C5H6O4 | [M-H]- | 129.019 | 129.0197 | 3.1 |
| 135 | Oxaloglutarate | 3.33 | C7H8O7 | [M-H]- | 203.02 | 203.0201 | 1.8 |
| 136 | N(alpha)-gamma-L-Glutamylhistamine | 5.25 | C10H16N4O3 | [M+AcO-H]- | 299.136 | 299.1365 | 1.5 |
| 137 | Homocarnosine | 5.25 | C10H16N4O3 | [M+AcO-H]- | 299.136 | 299.1365 | 1.5 |
| 138 | Glycochenodeoxycholate | 7.93 | C26H43NO5 | [M+Cl]- | 484.284 | 484.2818 | -3.7 |
| 139 | Taurolithocholate | 7.91 | C26H45NO5S | [M-H]- | 482.295 | 482.2931 | -3.1 |
| 140 | Arachidonate | 5.11 | C20H32O2 | [M-H]- | 303.233 | 303.2321 | -2.9 |
| 141 | (R)(-)-Allantoin | 2.98 | C4H6N4O3 | [M+Cl]- | 193.013 | 193.0129 | -2.5 |
| 142 | Acetylenedicarboxylate | 1.87 | C4H2O4 | [M-H]- | 112.988 | 112.9882 | 1.6 |
| 143 | N-Carbamyl-L-glutamate | 3.92 | C6H10N2O5NH3 | [M+Cl]- | 242.055 | 242.054 | -4 |
| 144 | Precorrin 5 | 7.78 | C45H54N4O17 | [M-2H]2- | 460.167 | 460.1672 | 0.6 |
| 145 | D-Glucuronate | 2.94 | C6H10O7 | [M-H]- | 193.035 | 193.0355 | 0.4 |
| 146 | NA | 3.11 | C5H11NO4S | [M-H]- | 180.034 | 180.0342 | 3.6 |
| 147 | UMP | 5.62 | C9H13N2O9P | [M+Na-2H]- | 345.011 | 345.0096 | -2.8 |
| 148 | alpha-D-Lyxose | 2.52 | C5H10O5 | [M-H]- | 149.046 | 149.0454 | -0.8 |
| 149 | 4-Hydroxy-2-oxopentanoate | 2.21 | C5H8O4 | [M-H]- | 131.035 | 131.0355 | 3.7 |
| 150 | L-2-Aminoadipate | 2.45 | C6H11NO4 | [M-H]- | 160.062 | 160.0617 | 1 |
| 151 | Hydantoin-5-propionate | 3.28 | C6H8N2O4 | [M-H]- | 171.041 | 171.0411 | -0.2 |
| 152 | (6Z,9Z,12Z)-Octadecatrienoic acid | 4.71 | C18H30O2 | [M-H]- | 277.217 | 277.2175 | 0.8 |
| 153 | 6-Hydroxy-3-succinoylpyridine | 3.16 | C9H9NO4 | [M-H]- | 194.046 | 194.0452 | -3.3 |
| 154 | N-Succinyl-L-glutamate | 4.29 | C9H13NO7NH3 | [M-H]- | 263.088 | 263.0897 | 4.5 |
| 155 | 2-Quinolinecarboxylic acid | 3.35 | C10H7NO2NH3 | [M-H]- | 189.067 | 189.0669 | -0.1 |
| 156 | Salicyluric acid | 3.16 | C9H9NO4 | [M-H]- | 194.046 | 194.0452 | -3.3 |
| 157 | Betalamic acid | 3.4 | C9H9NO5 | [M-H2O-H]- | 192.03 | 192.031 | 4.3 |
| 158 | D-Arabinono-1,4-lactone | 2.16 | C5H8O5 | [M-H2O-H]- | 129.019 | 129.0197 | 3.1 |
| 159 | Biocytin | 0.83 | C16H28N4O4S | [M-H]- | 371.176 | 371.1743 | -4.2 |
| 160 | Taurocholic acid | 6.33 | C26H45NO7S | [M-H]- | 514.284 | 514.2842 | -0.5 |
| 161 | 3-Methyl-2-oxovaleric acid | 1.19 | C6H10O3 | [M-H]- | 129.056 | 129.0562 | 3.5 |
| 162 | L-Cysteine | 4.12 | C3H7NO2S | [2M-H]- | 241.032 | 241.0313 | -3.9 |
| 163 | D-Octopine | 4.41 | C9H18N4O4 | [M-H]- | 245.126 | 245.1254 | -0.5 |
| 164 | (3E)-4-(2-Carboxyphenyl)-2-oxobut-3-enoate | 4.82 | C11H8O5 | [M+AcO-H]- | 279.051 | 279.0497 | -4.8 |
| 165 | Purine | 1.61 | C5H4N4 | [M-H]- | 119.036 | 119.036 | -2.9 |
| 166 | L-Lysine 1,6-lactam | 2.7 | C6H12N2O | [M+Cl]- | 163.064 | 163.0643 | -0.6 |
| 167 | Hexadecanoic acid | 4.26 | C16H32O2 | [M-H]- | 255.233 | 255.2325 | -1.9 |
| 168 | gamma-Glutamyltyramine | 5.12 | C13H18N2O4 | [M+Na-2H]- | 287.101 | 287.101 | -1.2 |
| 169 | Phosphonoacetaldehyde | 2.37 | C2H5O4PNH3 | [M-H]- | 140.012 | 140.0117 | -0.6 |
| 170 | 2-Oxoadipate | 2.36 | C6H8O5 | [M-H2O-H]- | 141.019 | 141.0199 | 4.1 |
| 171 | Citrate | 2.86 | C6H8O7 | [M-H2O-H]- | 173.009 | 173.009 | -1.1 |
| 172 | Fumaric acid | 1.02 | C4H4O4 | [M-H]- | 115.004 | 115.0041 | 3.5 |
| 173 | 5-L-Glutamyl-taurine | 3.92 | C7H14N2O6S | [M-H]- | 253.05 | 253.0507 | 3 |
| 174 | 5-Hydroxyindoleacetate | 3 | C10H9NO3 | [M-H]- | 190.051 | 190.0511 | 0.7 |
| 175 | Hypoxanthine 1 | 2.4 | C5H4N4O | [M-H]- | 135.031 | 135.0312 | -0.2 |
| 176 | D-Glucosamine | 4.16 | C6H13NO5NH3 | [M+Cl]- | 231.075 | 231.0745 | -3.6 |
| 177 | Benzylsuccinate | 3.63 | C11H12O4 | [M-H]- | 207.066 | 207.0661 | -0.8 |
| 178 | 3-Hydroxy-L-kynurenine | 2.03 | C10H12N2O4 | [M-2H]2- | 111.033 | 111.0324 | -1.6 |
| 179 | S-(Indolylmethylthiohydroximoyl)-L-cysteine | 5.33 | C13H15N3O3S | [M+Na-2H]- | 314.058 | 314.059 | 3 |
| 180 | (S)-Malate | 3.04 | C4H6O5CH3CN | [M-H]- | 174.041 | 174.0409 | 0.9 |
| 181 | Leukotriene D4 | 8.08 | C25H40N2O6SNH3 | [M-H]- | 512.28 | 512.2811 | 2.1 |
| 182 | Succinate | 1.69 | C4H6O4 | [M-H2O-H]- | 99.009 | 99.0093 | 5 |
