# Supplementary material for: Insights into the molecular basis of tick-borne encephalitis from multiplatform metabolomics
Source: PLoS Negl Trop Dis. 2021 Mar 10;15(3):e0009172. doi: 10.1371/journal.pntd.0009172 (PMC7984639; doi:10.1371/journal.pntd.0009172)
Supplement: S6 Table — MSI Level 2: Metabolites identified by LC-MS and spectrum similarity with public/commercial spectrum libraries. (DOCX) [file pntd.0009172.s006.docx]

| **NO.** | **Name** | **Chemical Formula** | **t-value** | **DB identifier** | **Control group to Patients group** | |
| --- | --- | --- | --- | --- | --- | --- |
|  |  |  |  |  | **p-value** | **Fold change** |
| **MSI level 2** | | | | | | |
| 1 | 9-cis,11-trans-Octadecadienoate | C_18_H_32_O_2_ | 27.17 | HMDB03797 | 2.84E-44 | 62.16 |
| 2 | Oleamide | C_18_H_35_NO | 25.42 | HMDB0002117 | 4.87E-42 | 60.29 |
| 3 | gamma-L-Glutamyl-S-(hercyn-2-yl)-L-cysteine S-oxide | C17H28N5O8S | 2.57 | C20995 | 1.18E-02 | 52.96 |
| 4 | 2-(Acetamidomethylene)succinate | C7H9NO5 | 2.22 | KEGGC01215 | 2.91E-02 | 35.83 |
| 5 | 9(10)-EpOME | C18H32O3 | 16.96 | KEGGC14825 | 2.52E-29 | 10.88 |
| 6 | beta-Alanyl-L-arginine | C_9_H_19_N_5_O_3_ | 12.42 | HMDB0060441 | 4.69E-21 | 9.90 |
| 7 | 4-Chlorophenoxyacetate | C8H7ClO3 | 10.08 | KEGGC07088 | 2.75E-16 | 9.45 |
| 8 | 10,16-Dihydroxyhexadecanoic acid | C_16_H_32_O_4_ | 16.96 | HMDB0037798 | 2.55E-29 | 9.12 |
| 9 | Bilirubin | C_33_H_36_N_4_O_6_ | 10.41 | HMDB0000054 | 5.96E-17 | 8.60 |
| 10 | 9,10-DHOME | C_18_H_34_O_4_ | 18.63 | HMDB0004704 | 4.16E-32 | 8.57 |
| 11 | 16-Oxo-palmitate |  | 16.65 |  | 8.70E-29 | 8.13 |
| 12 | S-Sulfo-L-cysteine | C3H7NO5S2 | 13.31 | KEGGC05824 | 8.80E-23 | 7.77 |
| 13 | Sinapine | C_16_H_24_NO_5_ | 2.91 | HMDB0029379 | 4.55E-03 | 7.71 |
| 14 | N-Acetylornithine | C7H14N2O3 | 7.18 | KEGGC00437 | 2.27E-10 | 7.00 |
| 15 | Glycocholate | C_26_H_42_NO_6_ | 16.27 | KEGGC01921 | 4.07E-28 | 6.06 |
| 16 | Leukotriene A4 | C_20_H_30_O_3_ | 14.06 | KEGGC00909 | 4.10E-24 | 6.01 |
| 17 | Phosphatidylethanolamine | C_41_H_82_NO_8_P | 2.22 | HMDB0060501 | 2.88E-02 | 5.92 |
| 18 | 3alpha,12alpha-Dihydroxy-5beta-chol-6-enoate | C_27_H_46_O_5_ | 16.48 | HMDB0060137 | 1.73E-28 | 5.90 |
| 19 | (2R,3R)-3-Methylornithinyl-N6-lysine | C12H26N4O3 | 10.18 | KEGGC20278 | 1.77E-16 | 5.78 |
| 20 | gamma-L-Glutamyl-L-cysteinyl-beta-alanine |  | 5.52 |  | 3.40E-07 | 5.74 |
| 21 | Latia luciferin |  | 15.33 |  | 1.86E-26 | 5.57 |
| 22 | Prostaglandin F2alpha | C_20_H_34_O_5_ | 9.24 | KEGGC00639 | 1.47E-14 | 5.30 |
| 23 | Pidolic acid | C_5_H_7_NO_3_ | 2.31 | HMDB0000267 | 2.32E-02 | 5.21 |
| 24 | 5-Hydroxy-L-tryptophan | C11H12N2O3 | 9.62 | KEGGC00643 | 2.15E-15 | 5.18 |
| 25 | L-Aspartyl-L-phenylalanine | C_13_H_16_N_2_O_5_ | 9.14 | HMDB0000706 | 2.32E-14 | 4.71 |
| 26 | (S)-Norcoclaurine | C16H17NO3 | 13.10 | C06160 | 2.69E-22 | 4.47 |
| 27 | 4-Hydroxy-2-quinolinecarboxylic acid | C_10_H_7_NO_3_ | 2.13 | HMDB0000715 | 3.57E-02 | 4.36 |
| 28 | Glycochenodeoxycholate | C_26_H_43_NO_5_ | 11.41 | KEGG C05466 | 5.61E-19 | 3.91 |
| 29 | 2-Succinylbenzoyl-CoA | C32H44N7O20P3S | 10.82 | C03160 | 8.66E-18 | 3.66 |
| 30 | Anthraniloyl-CoA | C28H41N8O17P3S | 10.39 | C02447 | 6.56E-17 | 3.62 |
| 31 | 3alpha,7alpha-Dihydroxy-12-oxo-5beta-cholanate | C_24_H_38_O_5_ | 10.12 | HMDB0000400 | 2.34E-16 | 3.52 |
| 32 | D-Arabinose 5-phosphate | C5H11O8P | 4.14 | KEGGC01112 | 8.12E-05 | 3.39 |
| 33 | Apigenin | C15H10O5 | 7.36 | KEGGC01477 | 9.67E-11 | 3.25 |
| 34 | Taurocyamine | C3H9N3O3S | 2.31 | C01959 | 2.31E-02 | 3.14 |
| 35 | 1-Deoxy-D-altro-heptulose 7-phosphate |  | 5.22 |  | 1.18E-06 | 3.13 |
| 36 | Pyroglutamic acid | C_5_H_7_NO_3_ | 3.09 | HMDB0000267 | 2.68E-03 | 3.09 |
| 37 | Pyrrolidonecarboxylic acid | HMDB0000267 | 3.09 | HMDB0000267 | 2.68E-03 | 3.09 |
| 38 | 6-Phospho-D-gluconate | C6H13O10P | 7.36 | C00345 | 9.54E-11 | 3.03 |
| 39 | 3-Deoxy-D-manno-octulosonate | C17H26N3O15P | 7.46 | C04121 | 6.22E-11 | 2.90 |
| 40 | Sphingosine | C_18_H_37_NO_2_ | 4.89 | HMDB0000252 | 4.49E-06 | 2.77 |
| 41 | Alpha-ketoisovaleric acid | C_5_H_8_O_3_ | 9.30 | HMDB0000019 | 1.07E-14 | 2.76 |
| 42 | 5-Hydroxy-2-oxo-4-ureido-2,5-dihydro-1H-imidazole-5-carboxylate | C5H6N4O5 | 5.98 | C12248 | 4.77E-08 | 2.73 |
| 43 | 4-Carboxy-4-hydroxy-2-oxoadipate | C7H8O8 | 2.73 | C04115 | 7.73E-03 | 2.60 |
| 44 | L-Glutamate | C_5_H_9_NO_4_ | 7.97 | HMDB0060475 | 5.34E-12 | 2.25 |
| 45 | D-Glutamine | C_5_H_10_N_2_O_3_ | 10.43 | HMDB0003423 | 5.28E-17 | 2.25 |
| 46 | L-Glutamine | C_5_H_10_N_2_O_3_ | 10.43 | HMDB0003423 | 5.28E-17 | 2.25 |
| 47 | Pantothenic Acid | C_9_H_17_NO_5_ | 5.02 | HMDB0000210 | 2.63E-06 | 2.25 |
| 48 | D-allo-Isoleucine |  | 2.26 |  | 2.64E-02 | 2.10 |
| 49 | Sphinganine 1-phosphate | C_18_H_40_NO_5_P | 6.86 | HMDB0001383 | 9.51E-10 | 2.03 |
| 50 | (Z)-Phenylacetaldehyde oxime | C8H9NO | 4.55 | C16075 | 1.72E-05 | 2.01 |
| 51 | L-Pipecolate | C6H11NO2 | 3.43 | C00408 | 9.10E-04 | 1.96 |
| 52 | O-Phospho-L-serine | C3H8NO6P | 4.70 | C01005 | 9.73E-06 | 1.96 |
| 53 | Sphingosine 1-phosphate | C18H38NO5P | 7.10 | C06124 | 3.17E-10 | 1.95 |
| 54 | L-2-Amino-3-oxobutanoic acid | C4H7NO3 | 3.34 | C03508 | 1.22E-03 | 1.94 |
| 55 | Glycerophosphocholine | C_8_H_20_NO_6_P | 6.39 | HMDB0000086 | 7.62E-09 | 1.90 |
| 56 | Indoxyl | C_8_H_7_NO | 4.64 | HMDB0004094 | 1.20E-05 | 1.82 |
| 57 | 1-Aminocyclopropane-1-carboxylate | C4H7NO2 | 6.43 | C01234 | 6.43E-09 | 1.81 |
| 58 | Carboxynorspermidine | C7H17N3O2 | 2.28 | C18174 | 2.49E-02 | 1.81 |
| 59 | Cholic acid | C24H40O5 | 2.33 | C00695 | 2.21E-02 | 1.79 |
| 60 | 8(R)-HPODE | C18H32O4 | 2.00 | C14831 | 4.89E-02 | 1.78 |
| 61 | 10-Formyldihydrofolate | C_20_H_21_N_7_O_7_ | 5.21 | HMDB0006485 | 1.25E-06 | 1.77 |
| 62 | L-Cystine | C_6_H_12_N_2_O_4_S_2_ | 11.27 | HMDB0000192 | 9.13E-19 | 1.76 |
| 63 | Hypoxanthine | C_5_H_4_N_4_O | 3.29 | HMDB0000157 | 1.42E-03 | 1.75 |
| 64 | Octadecanal |  | 2.82 |  | 5.97E-03 | 1.75 |
| 65 | L-Histidine | C6H14N4O2 | 2.28 | C00062 | 2.49E-02 | 1.75 |
| 66 | Taurine | C2H7NO3S | 5.68 | C00245 | 1.72E-07 | 1.73 |
| 67 | alpha-Tocopherol | C29H50O2 | 5.42 | C02477 | 5.11E-07 | 1.68 |
| 68 | L-Octanoylcarnitine | C_15_H_29_NO_4_ | 3.94 | HMDB0000791 | 1.60E-04 | 1.67 |
| 69 | 1,7-Dimethyluric acid | C_7_H_8_N_4_O_3_ | 3.81 | HMDB0011103 | 2.60E-04 | 1.66 |
| 70 | (Indol-3-yl)glycolaldehyde | C10H9NO2 | 2.41 | KEGGC03230 | 1.81E-02 | 1.64 |
| 71 | Xanthine | C_5_H_4_N_4_O_2_ | 5.58 | HMDB0000292 | 2.68E-07 | 1.63 |
| 72 | Ergothioneine | C_9_H_15_N_3_O_2_S | 3.13 | HMDB0003045 | 2.41E-03 | 1.63 |
| 73 | 3,4-Dihydroxyphenylacetate | C_8_H_8_O_4_ | 2.00 | HMDB0001336 | 4.89E-02 | 1.60 |
| 74 | 3,4-Dihydroxyfluorene | C13H10O2 | 3.51 | C07717 | 7.10E-04 | 1.60 |
| 75 | 3-Methyl-2-oxovaleric acid | C_6_H_10_O_3_ | 7.65 | HMDB0000491 | 2.54E-11 | 1.60 |
| 76 | (-)-Sedamine | C14H21NO | 2.80 | C10171 | 6.24E-03 | 1.58 |
| 77 | Pipecolic acid | C_6_H_11_NO_2_ | 3.15 | HMDB0000070 | 2.21E-03 | 1.58 |
| 78 | Dopamine | C_8_H_11_NO_2_ | 2.10 | HMDB0000073 | 3.82E-02 | 1.58 |
| 79 | D-Pipecolic acid | C_6_H_11_NO_2_ | 3.17 | HMDB0005960 | 2.11E-03 | 1.58 |
| 80 | 2,4-Dihydroxybenzoic acid | C_7_H_6_O_4_ | 2.67 | HMDB0029666 | 9.05E-03 | 1.57 |
| 81 | 2,6-Dihydroxybenzoic acid | C_7_H_6_O_4_ | 2.67 | HMDB0013676 | 9.05E-03 | 1.57 |
| 82 | sn-Glycero-3-phosphocholine |  | 3.11 |  | 2.54E-03 | 1.56 |
| 83 | 5-Methoxytryptamine | C_11_H_14_N_2_O | 4.62 | HMDB0004095 | 1.31E-05 | 1.53 |
| 84 | L-Asparagine | C_4_H_8_N_2_O_3_ | 4.35 | HMDB0000168 | 3.68E-05 | 1.52 |
| 85 | Chenodeoxycholate | C_24_H_39_O_4_ | 3.23 | C02528 | 1.75E-03 | 1.51 |
| 86 | 2-Hydroxypyridine | C_5_H_5_NO | 2.68 | HMDB0013751 | 8.79E-03 | 1.51 |
| 87 | N-Methylnicotinamide | C_7_H_8_N_2_O | 2.68 | HMDB0003152 | 8.81E-03 | 1.51 |
| 88 | Chloroacetic acid | C_2_H_3_ClO_2_ | 2.22 | HMDB0031331 | 2.90E-02 | 1.49 |
| 89 | ADP-ribose | C15H23N5O14P2 | 3.85 | C00301 | 2.20E-04 | 1.45 |
| 90 | Aminoacetone | C3H7NO | 2.26 | C01888 | 2.64E-02 | 1.44 |
| 91 | 4-Aminobutyraldehyde | C4H9NO | 2.36 | C00555 | 2.03E-02 | 1.42 |
| 92 | Ornithine | C5H12N2O2 | 3.55 | C00515 | 6.20E-04 | 1.39 |
| 93 | Perillic acid | C_10_H_14_O_2_ | 2.03 | HMDB0004586 | 4.53E-02 | 1.39 |
| 94 | Linoleate | C18H32O2 | 2.16 | C01595 | 3.34E-02 | 1.39 |
| 95 | Formaldehyde | CH_2_O | 2.98 | HMDB0001426 | 3.79E-03 | 1.38 |
| 96 | D-Ornithine | C_5_H_12_N_2_O_2_ | 3.43 | HMDB0003374 | 9.10E-04 | 1.38 |
| 97 | L-Ornithine | C6H14N2O2 | 3.43 | C00047 | 9.20E-04 | 1.38 |
| 98 | O-Succinyl-L-homoserine | C8H13NO6 | 2.75 | C01118 | 7.29E-03 | 1.37 |
| 99 | Choline phosphate |  | 6.58 |  | 3.32E-09 | 1.37 |
| 100 | Dehydroepiandrosterone sulfate | C_19_H_28_O_5_S | 2.43 | C04555 | 1.71E-02 | 1.36 |
| 101 | D-Alanyl-D-serine | C6H12N2O4 | 2.52 | C19719 | 1.37E-02 | 1.36 |
| 102 | 4-Ethylbenzoic acid | C_9_H_10_O_2_ | 2.86 | HMDB0002097 | 5.36E-03 | 1.36 |
| 103 | Sphingomyelin | C24H49N2O6PR | 2.37 | C00550 | 2.01E-02 | 1.36 |
| 104 | L-Methionine | C_5_H_11_NO_2_S | 5.92 | C00073 | 6.21E-08 | 1.35 |
| 105 | Trimethylamine N-oxide | C3H9NO | 2.08 | C01104 | 4.03E-02 | 1.34 |
| 106 | L-Threonine | C_4_H_9_NO_3_ | 3.68 | HMDB0000167 | 4.10E-04 | 1.33 |
| 107 | 1,3-Diaminopropane | C_3_H_10_N_2_ | 2.36 | HMDB0000002 | 2.06E-02 | 1.31 |
| 108 | Betaine | C_5_H_12_NO_2_ | 3.09 | HMDB0000043 | 2.73E-03 | 1.28 |
| 109 | Homo-L-arginine | C_7_H_16_N_4_O_2_ | 2.68 | HMDB00670 | 8.79E-03 | 1.28 |
| 110 | Ethylbenzene | C_8_H_10_ | 2.26 | HMDB0059905 | 2.60E-02 | 1.27 |
| 111 | Serotonin | C_10_H_12_N_2_O | 2.97 | HMDB0000259 | 3.87E-03 | 1.27 |
| 112 | N-Acetylputrescine | C_6_H_14_N_2_O | 2.55 | HMDB0002064 | 1.26E-02 | 1.26 |
| 113 | L-Acetylcarnitine | C_9_H_17_NO_4_ | 3.26 | HMDB0000201 | 1.59E-03 | 1.26 |
| 114 | O-Acetylcarnitine | C_9_H_18_NO_4_ | 3.26 | C02571 | 1.59E-03 | 1.26 |
| 115 | L-Proline | C_5_H_9_NO_2_ | 2.39 | HMDB0000162 | 1.92E-02 | 1.24 |
| 116 | Sarcosine | C_3_H_7_NO_2_ | 3.80 | HMDB0000271 | 2.70E-04 | 1.24 |
| 117 | Guanidinoacetate | C3H7N3O2 | 2.66 | C00581 | 9.32E-03 | 1.23 |
| 118 | Indolelactic acid | C_11_H_11_NO_3_ | 2.22 | HMDB0000671 | 2.94E-02 | 1.23 |
| 119 | Indolelactate |  | 2.21 |  | 2.95E-02 | 1.23 |
| 120 | L-Homoserine | C_4_H_9_NO_3_ | 3.34 | HMDB0000719 | 1.24E-03 | 1.22 |
| 121 | Thymine | C_5_H_6_N_2_O_2_ | 3.12 | HMDB0000262 | 2.45E-03 | 1.21 |
| 122 | Imidazoleacetic acid | C_5_H_6_N_2_O_2_ | 3.12 | HMDB0002024 | 2.45E-03 | 1.21 |
| 123 | D-Proline | C_5_H_9_NO_2_ | 2.63 | HMDB0003411 | 1.02E-02 | 1.20 |
| 124 | Psychosine | C24H47NO7 | 2.86 | C01747 | 5.33E-03 | 1.18 |
| 125 | Cortisone | C_21_H_28_O_5_ | 2.81 | HMDB02802 | 6.05E-03 | 1.18 |
| 126 | L-Serine | C_3_H_7_NO_3_ | 2.17 | HMDB0000187 | 3.29E-02 | 1.17 |
| 127 | Citric acid | C_6_H_8_O_7_ | 2.83 | HMDB0000094 | 5.75E-03 | 1.17 |
| 128 | 1-Methyladenosine | C_11_H_15_N_5_O_4_ | 2.78 | HMDB03331 | 6.69E-03 | 1.16 |
| 129 | Iminoglycine | C2H3NO2 | 2.33 | C15809 | 2.22E-02 | 1.15 |
| 130 | 3-Indoleacrylate | C_11_H_9_NO_2_ | 2.36 | HMDB0000734 | 2.07E-02 | 1.14 |
| 131 | Creatinine | C_4_H_7_N_3_O | 2.40 | HMDB0000562 | 1.84E-02 | 1.14 |
| 132 | D-Lysine | C_6_H_14_N_2_O_2_ | 2.19 | HMDB0003405 | 3.13E-02 | 1.13 |
| 133 | L-Lysine | C_6_H_14_N_2_O_2_ | 2.19 | HMDB0000182 | 3.13E-02 | 1.13 |
| 134 | L-Cysteate | C_3_H_7_NO_5_S | 2.05 | HMDB0002757 | 4.38E-02 | 1.13 |
| 135 | 2-Hydroxy-3-methylbenzalpyruvate | C11H10O4 | 2.89 | C14086 | 4.90E-03 | 1.13 |
| 136 | L-Tryptophan | C_11_H_12_N_2_O_2_ | 2.17 | HMDB0000929 | 3.25E-02 | 1.13 |
| 137 | Sucrose | C_12_H_22_O_11_ | 2.13 | HMDB0000258 | 3.58E-02 | 1.13 |
| 138 | Trehalose | C_12_H_22_O_11_ | 2.13 | HMDB0000975 | 3.58E-02 | 1.13 |
| 139 | CMP-2-aminoethylphosphonate | C_11_H_20_N_4_O_10_P_2_ | 2.08 | HMDB0060067 | 4.09E-02 | 1.12 |
| 140 | Indole | C_8_H_7_N | 2.36 | HMDB0000738 | 2.05E-02 | 1.12 |
| 141 | Indoleacrylic acid | C_11_H_9_NO_2_ | 2.39 | HMDB00734 | 1.91E-02 | 1.12 |
| 142 | Skatole | C9H9N | 2.38 | C08313 | 1.97E-02 | 1.12 |
| 143 | Uric acid | C_5_H_4_N_4_O_3_ | 2.34 | HMDB0000289 | 2.17E-02 | 1.10 |
| 144 | Urate |  | 2.03 |  | 4.50E-02 | 1.10 |
