# Supplementary figures and images for: Insights into the molecular basis of tick-borne encephalitis from multiplatform metabolomics
Source: PLoS Negl Trop Dis. 2021 Mar 10;15(3):e0009172. doi: 10.1371/journal.pntd.0009172 (PMC7984639; doi:10.1371/journal.pntd.0009172)

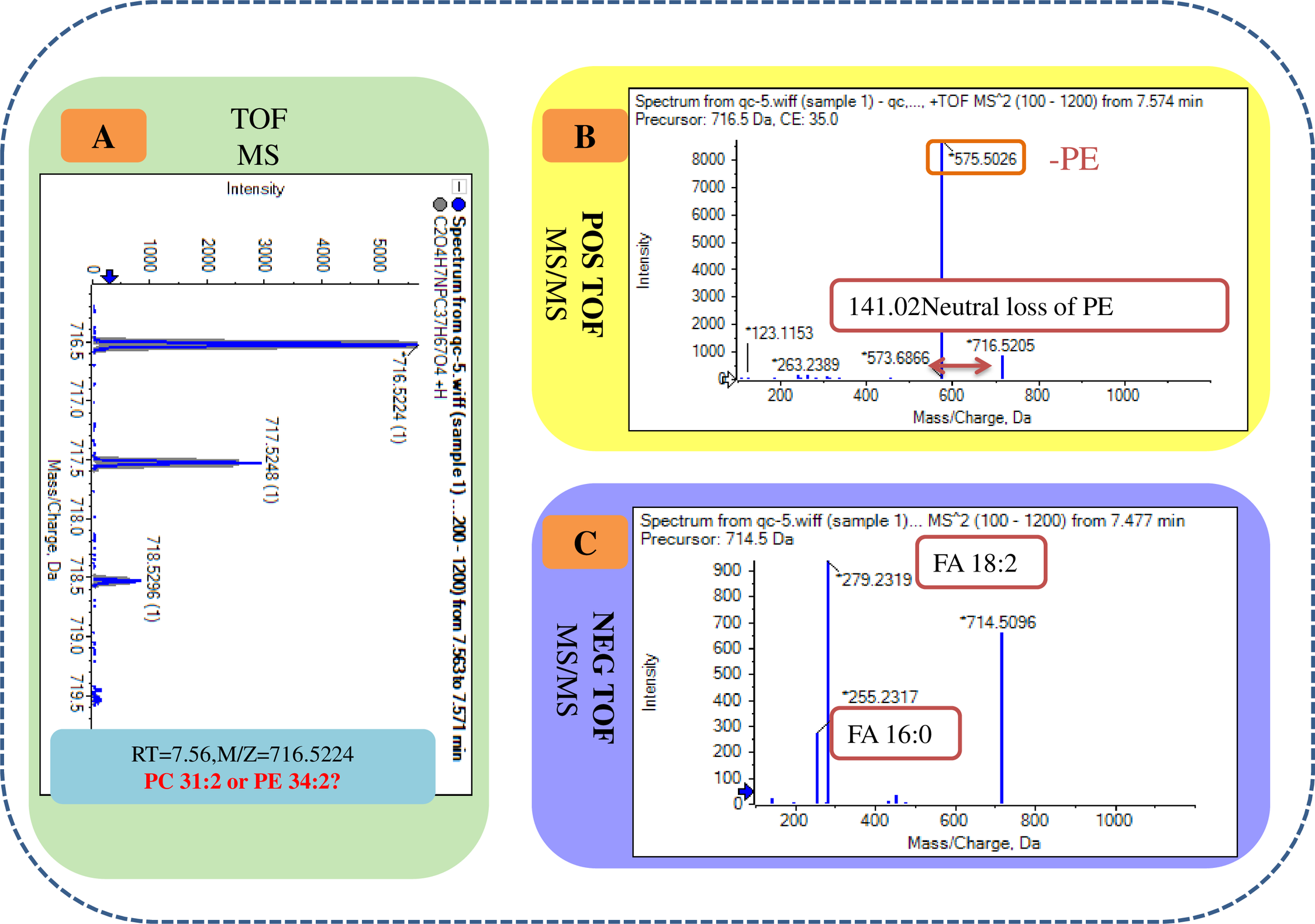

Supplement: S1 Fig — Example identification of PE 34:2 as a differential metabolite to expound the authentication process. A. Extracted ion chromatogram (EIC) and matched ion m/z 716.5224. B. MS/MS spectrum and proposed pathways in positive mode. C. MS/MS spectrum and proposed pathways in negative mode. (TIF) [file pntd.0009172.s007.tif]

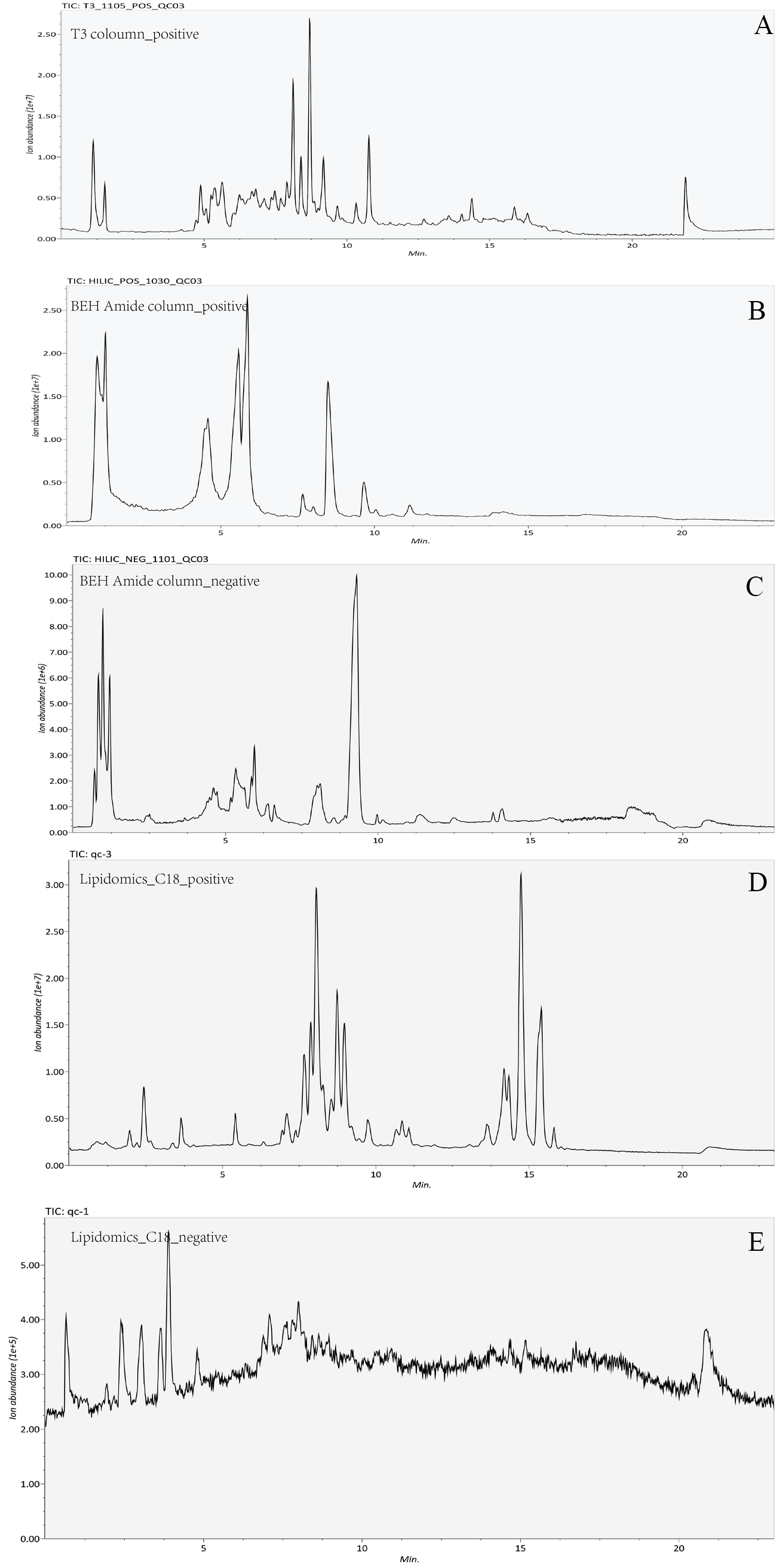

Supplement: S2 Fig — Chromatograms representing serum metabolic fingerprints of quality control (QC) samples obtained with LC-TOF-MS in (A) positive ionization mode on a T3 column, (B)positive mode on an amide column, (C) negative mode on an amide column and lipidomics in (D) positive mode and (E) negative mode. (TIF) [file pntd.0009172.s008.tif]

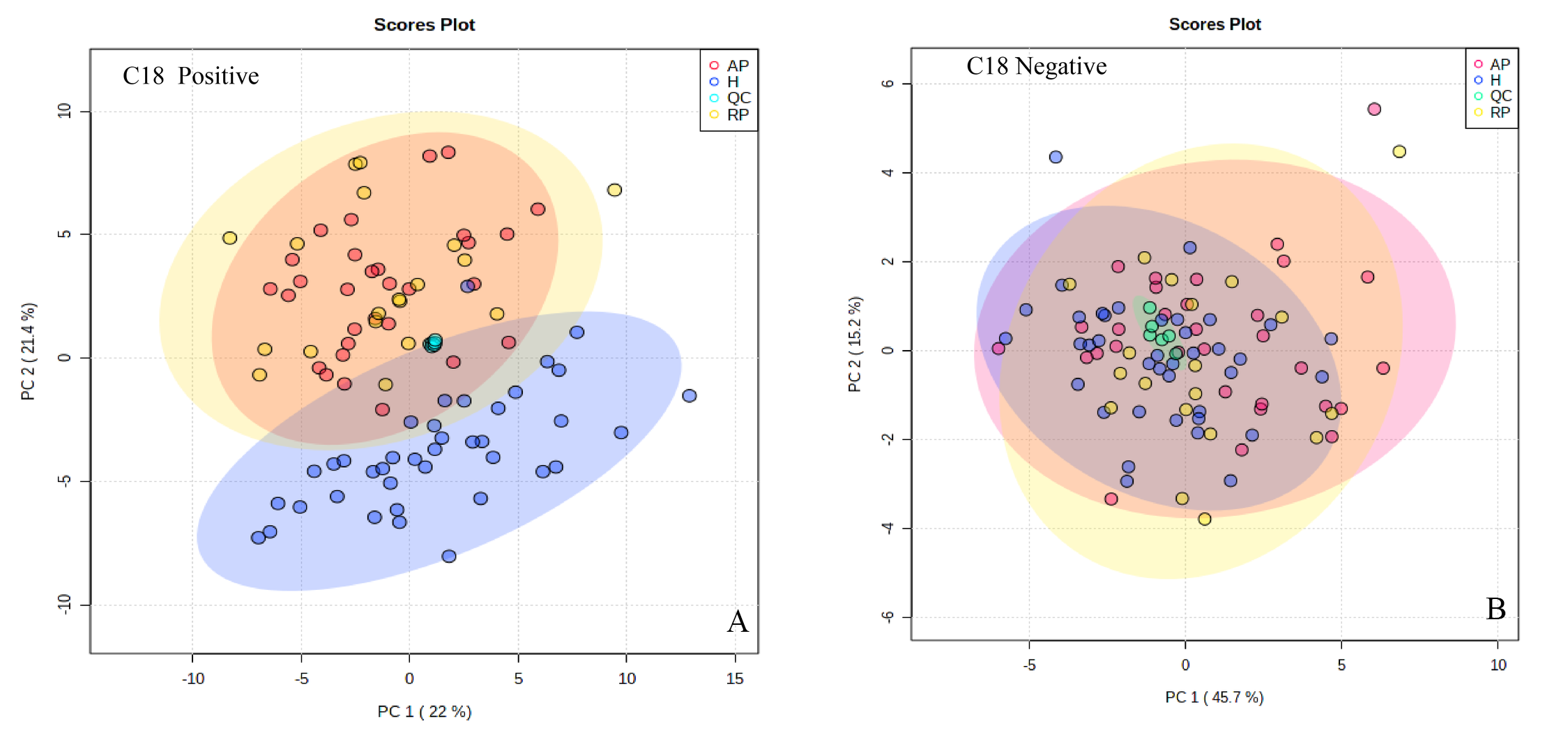

Supplement: S3 Fig — PCA models built on lipid data obtained with LC-ESI-TOF-MS analysis in (A) positive mode (PC1 = 22.7%, PC2 = 20.2%) and (B) negative mode (PC1 = 43.1%, PC2 = 14.1%). Red, purple, blue and yellow solid circles correspond to AP, healthy, QC and RP samples, respectively. (TIF) [file pntd.0009172.s009.tif]

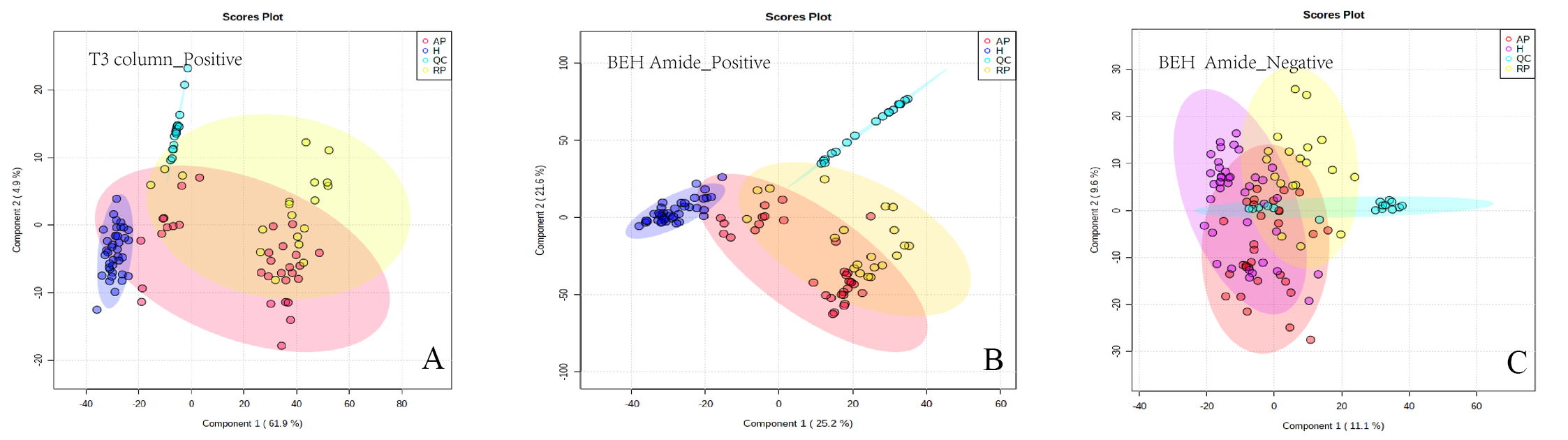

Supplement: S4 Fig — PLS models based on metabolomics data obtained with LC-ESI-TOF-MS analysis in (A) positive mode on a T3 column, (B) positive mode on amide column and (C) negative mode on amide column. Red, purple, blue and yellow solid circles correspond to AP, healthy, QC and RP samples, respectively. (TIF) [file pntd.0009172.s010.tif]

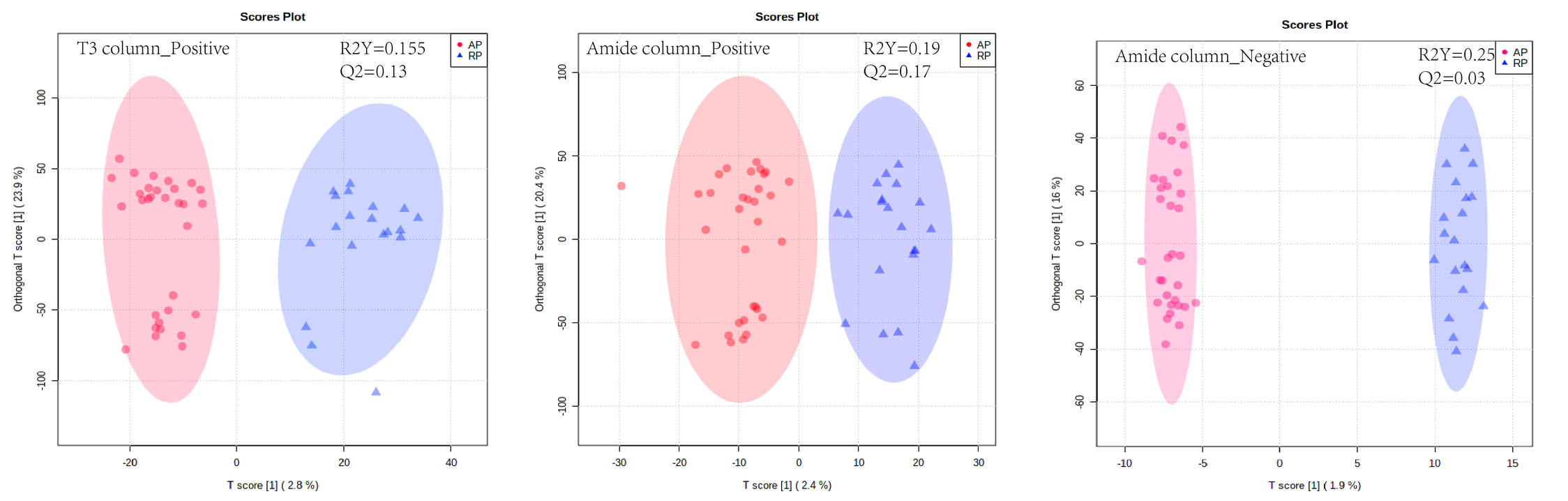

Supplement: S5 Fig — The OPLS-DA models were constructed using LC-MS/MS data from acute phase and recovery phase TBEV patients on a T3 column and an amide column. (TIF) [file pntd.0009172.s011.tif]

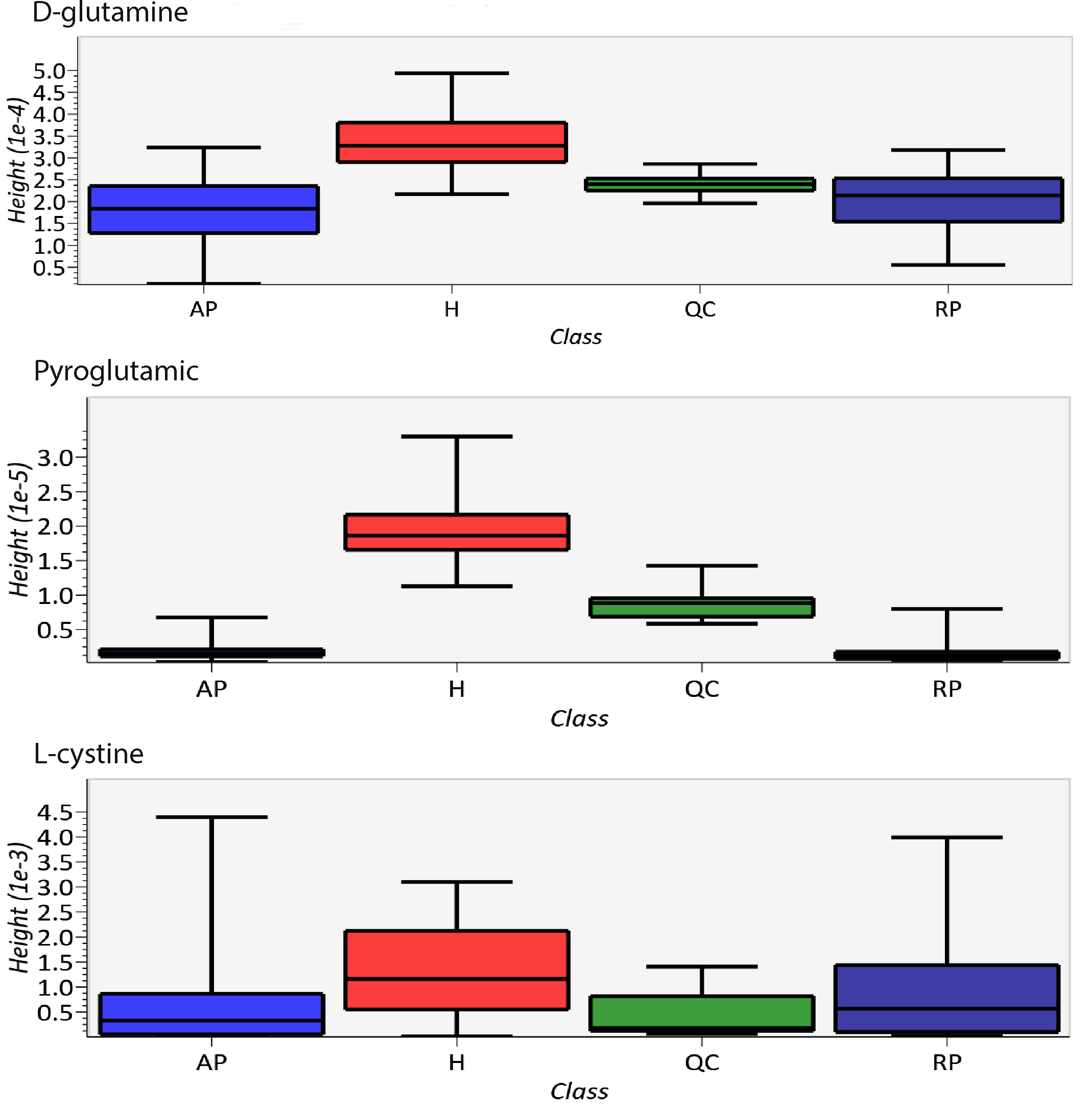

Supplement: S6 Fig — (Red box: healthy samples; green box: QC samples; blue box: acute phase patients; Modena box: recovery phase patients). (TIF) [file pntd.0009172.s012.tif]

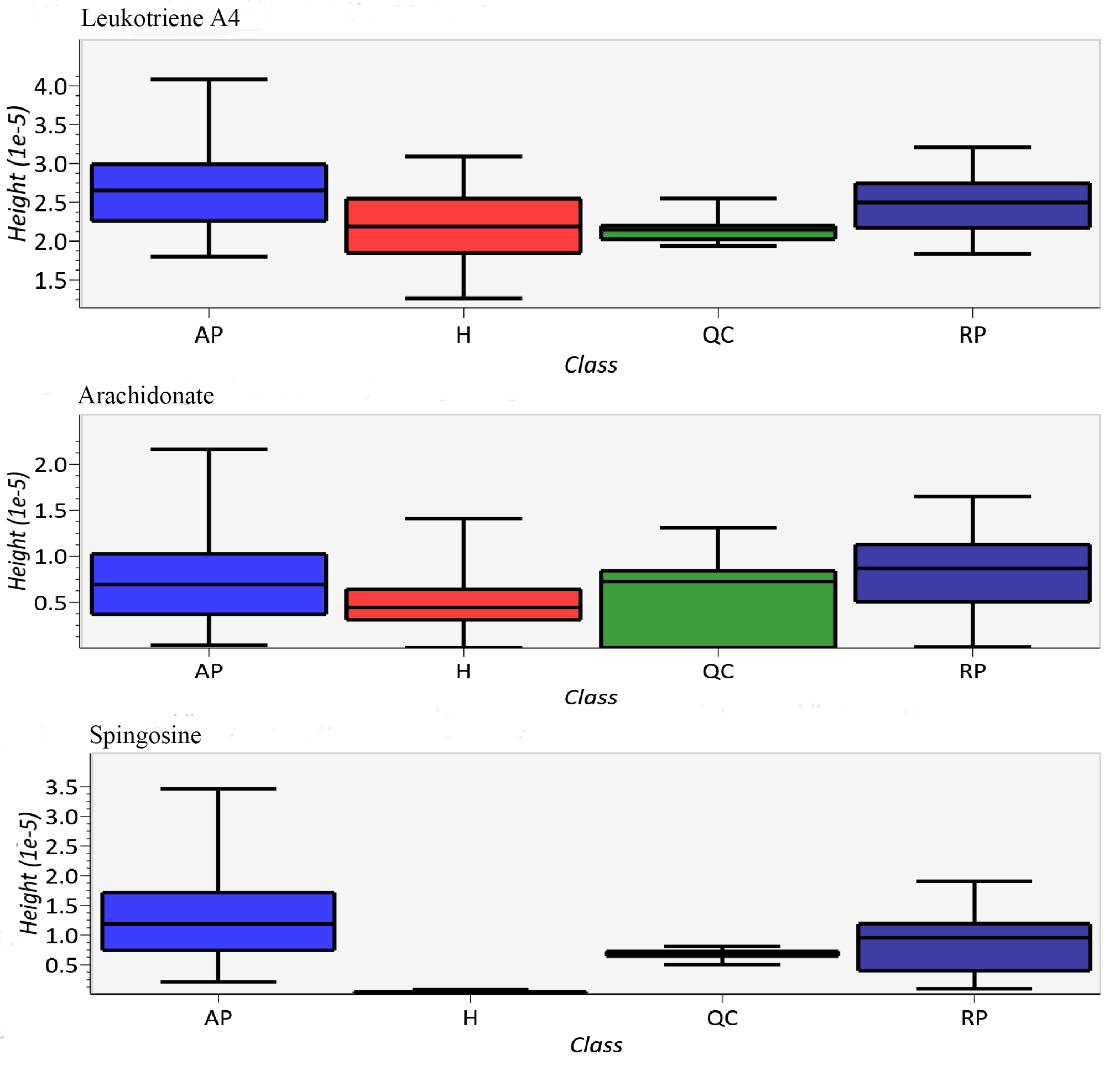

Supplement: S7 Fig — (Red box: healthy samples; green box: QC samples; blue box: acute phase patients; Modena box: recovery phase patients (TIF) [file pntd.0009172.s013.tif]
