# Supplementary material for: Insights into the molecular basis of tick-borne encephalitis from multiplatform metabolomics
Source: PLoS Negl Trop Dis. 2021 Mar 10;15(3):e0009172. doi: 10.1371/journal.pntd.0009172 (PMC7984639; doi:10.1371/journal.pntd.0009172)
Supplement: Attachment — Submitted filename: Reviewer_aug02_20.pdf [file pntd.0009172.s014.pdf]

Review of the paper "Insights into the molecular basis of tick-borne encephalitis from Multiplatform Metabolomics"

Aug 02, 2020

General comments. The written of the manuscript needs to be revised. There are problems with misspellings, lack of punctuation, and sentences that need to be rephrased. Also, some scientific terms are not used correctly. The hypothesis and objectives of the study are not clear. The main concern is the lack of information in the methodology and some results that are missing. References are also missing.

Abstract

Line 21. Needs to be rewritten.

Line 23. The authors claimed that lipid and metabolic changes were assessed during disease progression. However, to achieve this purpose they would need to perform a prospective study. Same thing in line 29.

Introduction

Line 77. The sentence is not clear.

Line 86-87. The sentence is confusing. Please rewrite.

Line 96. The hypothesis of the study needs to be stated and how the study design will address this hypothesis.

Line 97. Replace "acquision modes" by approaches.

Lines 97-98. The current methodology and findings do not allow the authors to "provide insight into tick biology and pathogen transmission". The authors need to state the objectives and hypotheses of the study.

Methods.

General comments. Several methodological information regards the metabolomics/lipidomics analysis was missing.

Line 110. They mentioned that the final classification of TBEV was based on clinical test results and symptoms. What were the clinical tests and symptoms considered for the classification? A dot is missing.

Line 111. What were the other criteria used to classify a patient as AP?

Line 114. Aspirate transaminase??

Line 135. Were all peaks presented a relative standard deviation (RSD) below 10%? This is unlikely to happen considering that signal intensity of many peaks can drift from day to day and sometimes from run to run. Also, the occurrence of missing values is something

that also occurs and contributes to a higher variance in the data. Please, clarify. Were these peaks from the QC injection?

Line 145-146. References for XCMS and Metaboanalyst are required. Which XCMS was used (XCMS online or the XCMS package in R)? Please, mention the version of XCMS and metaboanalyst used. XCMS and Metaboanalyst were used to find the metabolites that differed significantly in TBE patients. Was it XCMS used only to process raw spectra? Was Metaboanalyst used only for statistical analysis? Additional details of data processing as well as of the statistical analysis are required for a better comprehension of the metabolomics/lipidomics analysis. What was the criterion used to filter the features? What was the normalization approach used for metabolomics and lipidomics? Moreover, the authors need to inform which algorithm and parameters were used for peak detection (*m/z* deviation; chromatographic peak width range; signal-to-noise ratio threshold; etc.). Same for retention-time correction and chromatogram alignment. How were the missing values handled?? All these procedures are crucial to remove bias introduced by LC-MS approach, such as outlier runs or peaks, removal of noise and contaminants, reduction the systematic variation of LC-MS data.

Line 147-148. OPLS-DA is a supervised not an unsupervised method.

Line 151. Please indicate when the student t-test and Mann Whitney were used. Each statistical test works with different assumptions and it is not common to use both tests for the same data. The authors need to establish their questions and assumptions about the data first and then choose the statistical test of interest. Also, it is crucial to correct for multiple comparisons to avoid false positives (e.g., false discovery rate). How the QCs samples were run? How many times was the QC sample injected?

Line 158. Which established strategy? Cite the references for this strategy and also explain it. In the text, the authors should mention that the identification obtained was a putative identification since no authentic standards were used.

Line 165. Further details are required to understand how the pathways analysis and the interaction networks were performed. In the pathway analysis, what were the criteria to consider a metabolic pathway as altered?

Results.

General comments. Indicate in the figures and tables which statistical test was used. The amount of features obtained for each LC-MS experiment should also be described in the results as well as the number of features retained after all the filtering process. Be clear in the text that these compounds are putative identified since no authentic standards were used to confirm the structures of the compounds.

Figure 1. The figure shows the PCA from features obtained in positive mode. What about the negative mode since in the methodology the authors say that the data was acquired in negative and positive mode.

Figure 2a. I suggest using numbers or letters to indicate in the legend the name of the

metabolic pathway instead writing the name of the pathway in the graph.

Line 183. To determine the flux of a metabolite it is needed the use not only of metabolomics approaches but also computational modeling of pathways. Thus the authors cannot claim that they checked the metabolite flux.

Table 2. It seems that authors transformed the abundance values to log2. However this was not mentioned in the methodology. Please clarify.

Figure 3. The letters are too small in the x and y axis. Also, what are the letters a, b ,c d, e and f?

Discussion.

General comments. How the metabolic findings could be potentially linked with immune responses observed in TBEV patients.

Line 242. Metabomic??

Lines 323 – 324. The results of the study do not allow such conclusion. The metabolic process that occurs during infectious diseases are too complex to establish a cause/consequence link between the metabolic changes and the disease.
