# Supplementary material for: Insights into the molecular basis of tick-borne encephalitis from multiplatform metabolomics
Source: PLoS Negl Trop Dis. 2021 Mar 10;15(3):e0009172. doi: 10.1371/journal.pntd.0009172 (PMC7984639; doi:10.1371/journal.pntd.0009172)
Supplement: Attachment — Submitted filename: Response letter.docx [file pntd.0009172.s015.docx]

Dear Alain Kohl:

Thank you very much for giving us an opportunity to revise our manuscript. We appreciate the editor and reviewers very much for their constructive comments and suggestions on our manuscript entitled “Insights into the Molecular Basis of Tick-borne Encephalitis from Multiplatform Metabolomics”.

**Abstract**

**Q:** Line 21. Needs to be rewritten.

R: The sentence had been rewritten.

**Q:** The authors claimed that lipid and metabolic changes were assessed during disease progression. However, to achieve this purpose they would need to perform a prospective study. Same thing in line 29.

**R:** Disease progression here refers to the infected patient. Though we try to discover the difference between the AP and the RP, there is no obvious separation owing to the limiting factor.

**Introduction**

**Q:** Line 77. The sentence is not clear.

**R:** The sentence had been rewritten.

**Q：**Line 86-87. The sentence is confusing. Please rewrite.

**R:** The sentence had been rewritten

**Q:** Line 96. The hypothesis of the study needs to be stated and how the study design will address this hypothesis.

**R:** We had provided supplementary data.

**Q：**Line 97. Replace “acquision modes” by approaches.

**R：** “Acquision modes” had been replaced to approaches.

**Q:** Lines 97-98.The current methodology and findings do not allow the authors to“provide insight into tick biology and pathogen transmission”. The authors need to state the objectives and hypotheses of the study.

**R:** We restated the objectives and hypotheses of the study.

**Methods**

**Q:** Line 110.They mentioned that the final classification of TBEV was based on clinical test results and symptoms. What were the clinical tests and symptoms considered for the classification? A dot is missing.

**R:** The information about the clinical tests and symptoms considered for the classification had been supplemented.

**Q:** Line 111.What were the other criteria used to classify a patient as AP?

**R:** The other criteria used to classify a patient as an AP had been supplemented.

**Q:** Line 114. Aspirate transaminase?

**R:** The contents related to aspirate transaminase had been deleted.

**Q:** Line 135.Were all peaks presented a relative standard deviation (RSD) below 10%? This is unlikely to happen considering that signal intensity of many peaks can drift from day to day and sometimes from run to run. Also, the occurrence of missing values is something that also occurs and contributes to a higher variance in the data. Please, clarify. Were these peaks from the QC injection?

**R:** The peaks presented a relative standard deviation (RSD) below 10% were QC samples. We use the RSD of QCs to assess the data quality.

**Q:** Line 145-146. References for XCMS and Metaboanalyst are required. Which XCMS was used (XCMS online or the XCMS package in R)? Please, mention the version of XCMS and metaboanalyst used. XCMS and Metaboanalyst were used to find the metabolites that differed significantly in TBE patients. Was it XCMS used only to process raw spectra? Was Metaboanalyst used only for statistical analysis? Additional details of data processing as well as of the statistical analysis are required for a better comprehension of the metabolomics/lipidomics analysis. What was the criterion used to filter the features? What was the normalization approach used for metabolomics and lipidomics? Moreover, the authors need to inform which algorithm and parameters were used for peak detection(m/z deviation; chromatographic peak width range; signal-to-noise ratio threshold; etc.). Same for retention-time correction and chromatogram alignment. How were the missing

values handled?? All these procedures are crucial to remove bias introduced by LC-MS approach, such as outlier runs or peaks, removal of noise and contaminants, reduction the systematic variation of LC-MS data.

**R:** The reference for XCMS and Metaboanalyst had been supplemented. The version of XCMS and Metaboanalyst had been added. The workflow and parameters of data processing had been illustrated in detail.

**Q:** Line 147-148. OPLS-DA is a supervised not an unsupervised method.

**R: “**Unsupervised” had been changed to “supervised”.

**Q:** Line 151. Please indicate when the student *t*-test and Mann Whitney were used. Each statistical test works with different assumptions and it is not common to use both tests for the same data. The authors need to establish their questions and assumptions about the data first and then choose the statistical test of interest. Also, it is crucial to correct for multiple comparisons to avoid false positives (e.g., false discovery rate). How the QCs samples were run? How many times was the QC sample injected?

**R:** The student t-test was used in data analysis. The injection batch of QC sample had been supplemented in the text. Six random control serum samples were injected at the start and end of each analytical batch to condition the analytical platform.

**Q:** Line 158. Which established strategy? Cite the references for this strategy and also explain it. In the text, the authors should mention that the identification obtained was a putative identification since no authentic standards were used.

**R:** The reference had been cited. And we also mentioned that the identification was a putative identification (MS2 level).

**Q:** Line 165. Further details are required to understand how the pathways analysis and the interaction networks were performed. In the pathway analysis, what were the criteria to consider a metabolic pathway as altered?

**R:** The details about the pathways analysis had been illustrated. The enrichment analysis was performed using a topology analysis that takes into account the positions of significant metabolites in metabolic pathways. Two indexes were estimated in the topology analysis: the centrality and the pathway impact. The centrality measures the number of shortest paths going through the target metabolite, which is employed to assess the significance of each metabolite within a specific pathway. The pathway impact is the cumulative value of the significant metabolites.

**Results**

**Q:** The figure shows the PCA from features obtained in positive mode. What about the negative mode since in the methodology the authors say that the data was acquired in negative and positive mode.

**R:** The PCA obtained in negative mode was supplemented, as shown in **Fig.1**.

**Q:** Figure 2a. I suggest using numbers or letters to indicate in the legend the name of the

metabolic pathway instead writing the name of the pathway in the graph.

**R:** I had made changes based on suggestions.

**Q:** Line 183. To determine the flux of a metabolite it is needed the use not only of metabolomics approaches but also computational modeling of pathways. Thus the authors cannot claim that they checked the metabolite flux.

**R:** “Flux” had changed to coverage.

**Q:** Table 2. It seems that authors transformed the abundance values to log2. However, this was not mentioned in the methodology. Please clarify**.**

**R:** We had clarified the transformation parameters of the abundance values.

**Q:** Figure 3. The letters are too small in the x and y axis. Also, what are the letters a, b ,c d, e

and f?

**R:** We had revised the Figure 3.

**Discussion**

**Q:** Line 242. Metabomic?

**R:** “Metabomic” had changed to “metabonomics”.

**Q:** Lines 323 – 324. The results of the study do not allow such conclusion. The metabolic process that occurs during infectious diseases are too complex to establish a cause/consequence link between the metabolic changes and the disease.

**R:** We supplemented the information in the methodology and the results. And we had advanced the quality for our figures. The hypothesis had been improved.

**R:** The validation of markers was a putative identification, not matching with the Standard Database. And the identification of PE is an example of mapping algorithm.

The English in this document has been checked by at least professional editors, both native speakers of English. For a certificate, please see: <http://www.textcheck.com/certificate/index/OVItKa> and <http://www.textcheck.com/certificate/index/p6oG1F>.
