# Supplementary material for: Insights into the molecular basis of tick-borne encephalitis from multiplatform metabolomics
Source: PLoS Negl Trop Dis. 2021 Mar 10;15(3):e0009172. doi: 10.1371/journal.pntd.0009172 (PMC7984639; doi:10.1371/journal.pntd.0009172)
Supplement: Attachment — Submitted filename: Response letter 1211.docx [file pntd.0009172.s016.docx]

Dear Alain Kohl:

Thank you very much for giving us an opportunity to revise our manuscript. We appreciate the editor and reviewers very much for their constructive comments and suggestions on our manuscript entitled “Insights into the Molecular Basis of Tick-borne Encephalitis from Multiplatform Metabolomics”.

**Q:** Please respond to the comments by reviewers 1 and 3 in particular, carefully. Clarity of figures and legends (figures 3/4) in particular should be improved.

**R**: Clarity of figures and legends (figures3/4) had been improved.

**Introduction**

**Q**：Reviewer #1: The hypothesis may still be improved. In this study the authors used several approaches to separate the metabolites according the polarity of the compounds. This indicates that part of their objectives is to have high coverage in the metabolomics study. This should be mentioned in the introduction to make the objective of the study more clear.

**R：**The sentence had been rewritten. We had improved the introduction to make the objective of the study more clearly.

**Methods**

**Q：**The sample size is sufficient. However, the amount of samples was not provided in the methodology but in the results section. This information should be in the methodology.

**R:** The information of samples in the methodology was shown in Table.S3.

**Q:** Did the authors performed correction for multiple hypothesis after the T-test? This is important to avoid false-positives.

**R:** We had performed correction for multiple hypothesis after the T-test to avoid false-positives.

**Q:** The authors are not clear about the amount of features in the QC that exhibit a relative standard deviation (RSD) lower than 10%. In the text, it seems that all features from the QCs injections exhibited a RSD<10%. Although, the majority of features could display a RSD<10% it is unlikely that all features presented such behavior. The authors should mentioned the percentage of features that presented a RSD<10%.

**R:** We submitted the data description and rewrote the sentence. In the experimental result, only a portion of features displayed a RSD<10%.

**Q:** The authors mentioned that features with a p<0.05 displayed a significant difference between the samples. However, was the fold change (or log2FC) used as criteria to classify a feature as significant? Please clarify the criteria used to claim that a feature was statistical significant.

**R:** The fold change (FC) threshold as a complementary criterion in metabolomics data evaluation was also used as an indicator to differentiate the groups.

**Results**

**Q:** Fig 3 right panel - is unreadable and not useful. Can you present that data using an alternative graphic - may be using more dimensions?

**R:** We had presented that data using an alternative graphic.

**Q**: Fig 4 - I did not find the figure legend. This figure legend needs to be very detailed to walk the reader through the hypotheses behind the pathway diagram. So while the figure is probably the most important take-away, some detailed descriptions are neccessary.

**R:** The figure legend had been implemented.

**Q:** Lines 184-185 - Please correct accuracy if sentence: “PCA scores representing TBEV samples and healthy samples were obtained using the T3 column and BEH Amide column” – the PCA scores were not obtained using the columns. It was obtained using the data generated from the methods using T3 and BEH columns.

**R:** The sentence had been rewritten.

**Conclusion**

**minor modifications**

**Q:** Lines 212-213. Is not correct to use the term expression when refer to a metabolite level. A metabolite is not expressed.

**R:** The sentence had been rewritten.

**Q:** In line 175, it was mentioned that “…The enrichment analysis was performed using a topology analysis that takes into account the positions of significant metabolites in metabolic pathways…” Actually, in the Metaboanalyst the pathway analysis combines enrichment and topology analysis to indicate which metabolic pathways are disturbed in a disease or experimental condition. Please rephrase this sentence to improve the explanation about pathway analysis in Metaboanalyst.

**R:** We had rephrased the sentence to improve the explanation about pathways analysis in Metaboanalyst.

**Q:** Line178. Which criteria in the pathway analysis were used to consider that a metabolic pathway was disturbed? Impact values closer to 1.0 indicate a more perturbed pathway and some works consider that when an impact value for a pathway is higher than 0.1 this indicate that the pathway is disturbed. Did the authors set a threshold for the pathway impact value?

**R:** We did not set a threshold for the pathway impact value. We only concentrated on pathways with higher impact value.
