# Supplementary material for: Insights into the molecular basis of tick-borne encephalitis from multiplatform metabolomics
Source: PLoS Negl Trop Dis. 2021 Mar 10;15(3):e0009172. doi: 10.1371/journal.pntd.0009172 (PMC7984639; doi:10.1371/journal.pntd.0009172)
Supplement: Attachment — Submitted filename: Response letter 0118.docx [file pntd.0009172.s017.docx]

Dear Alain Kohl:

Thank you very much for giving us an opportunity to revise our manuscript. We appreciate the editor and reviewers very much for their constructive comments and suggestions on our manuscript entitled “Insights into the Molecular Basis of Tick-borne Encephalitis from Multiplatform Metabolomics”.

**Q:** Tables should not be uploaded as individual files.  Please remove these files and include the tables in your manuscript file.

**R:** Tables as individual files are included in manuscript. Only tables supportive are uploaded as individual files as support information.

**Q：** Please see the attached iThenticate report and specifically the section highlighted in red in the results and discussion section which overlaps with the following source <https://journals.plos.org/plosntds/article?id=10.1371%2Fjournal.pntd.0002373>.
Can we please ask you to reword the highlighted section and add a citation to the reference list.

R: we had reworded the highlighted section and added a citation to the reference list.
